# Temporal dynamics of cognitive functioning in people with Parkinson’s disease

**DOI:** 10.1038/s41531-026-01338-3

**Published:** 2026-04-06

**Authors:** Daniel Scharfenberg, Elke Kalbe, Anja Ophey, Monika Balzer-Geldsetzer, Daniela Berg, Rüdiger Hilker-Roggendorf, Jan Kassubek, Inga Liepelt-Scarfone, Brit Mollenhauer, Kathrin Reetz, Oliver Riedel, Sandra Roeske, Jörg B. Schulz, Alexander Storch, Claudia Trenkwalder, Karsten Witt, Richard Dodel, Marie K. Deserno

**Affiliations:** 1 Medical Psychology | Neuropsychology and Gender Studies, Center for Neuropsychological Diagnostics and Intervention (CeNDI), University Hospital Cologne and Faculty of Medicine, University of Cologne, Cologne, Germany; 2 Ethikkommission, Ludwig-Maximilians-Universität München, Munich, Germany; 3 Department of Neurology, University Medical Center Schleswig-Holstein, Christian Albrechts-University (CAU), Campus Kiel, Kiel, Germany; 4 Department of Neurology, Klinikum Vest, Recklinghausen, Germany; 5 Ruhr-University, Bochum, Germany; 6 Department of Neurology, University Hospital Ulm, Ulm, Germany; 7 Department of Neurodegenerative Diseases, Hertie Institute for Clinical Brain Research and German Center for Neurodegenerative Diseases (DZNE), University Tübingen, and German Center for Neurodegenerative Diseases, Tübingen, Germany; 8 IB-Hochschule, Stuttgart, Germany; 9 Department of Neurology, University Medical Center, Göttingen, Germany; 10 Paracelsus-Elena Klinik, Kassel, Germany; 11 Department of Neurology, RWTH Aachen University Hospital, Aachen, Germany; 12 Molecular Neuroscience and Neuroimaging (INM-11), JARA Institute, Jülich, Germany; 13 Department Clinical Epidemiology, Leibniz Institute for Prevention Research and Epidemiology-BIPS, Bremen, Germany; 14 German Center for Neurodegenerative Diseases (DZNE), Bonn, Germany; 15 Department of Neurology, University of Rostock and German Center for Neurodegenerative Diseases (DZNE) Rostock/Greifswald, Rostock, Germany; 16 Department of Neurology, School of Medicine and Health Sciences and Research Center Neurosensory Science, University of Oldenburg, Oldenburg, Germany; 17 Department of Neurology, Evangelic Hospital Oldenburg, Oldenburg, Germany; 18 Center of Neurosensory Sciences, University of Oldenburg, Oldenburg, Germany; 19 Department of Geriatric Medicine, University Duisburg-Essen, Essen, Germany; 20 Clinical Psychology, Department of Psychology, University of Amsterdam, Amsterdam, the Netherlands

**Keywords:** Computational biology and bioinformatics, Diseases, Neurology, Neuroscience

## Abstract

Cognitive domains are central to diagnosing cognitive impairment in people with Parkinson’s disease (PwPD), yet are defined by expert consensus and cross-sectional data that assume temporal stability. This study examined whether cognitive domains remain stable or reorganize dynamically over time in PwPD. Using dynamic exploratory graph analysis, we analyzed 19 cognitive test scores from 355 PwPD across four yearly assessments (24,372 data points) from the DEMPARK/LANDSCAPE-study to identify dynamic organization of cognitive dimensions. Panel graphical vector autoregression models assessed dynamic couplings among dimensions. Five dynamic cognitive dimensions emerged, diverging substantially from theoretical domains and cross-sectional dimensions at baseline. Dynamic coupling revealed a temporal separation between card-sorting/cognitive flexibility and visuoconstruction. Cognitive domains in PwPD reorganize over time rather than remaining stable, challenging the static assumption of diagnostic criteria. Using PD as an exemplar, findings demonstrate the need for dynamic frameworks potentially revealing condition-specific temporal architectures when applied to other neurodegenerative disorders.

## Introduction

Understanding subtypes of cognitive impairment, i.e., single- or multiple domain mild cognitive impairment (MCI)^1^, is crucial for predicting further cognitive decline^2^ or to inform individualized cognitive interventions in neurodegenerative diseases such as Parkinson’s disease (PD)^3^. However, existing definitions of cognitive domains in different guidelines of cognitive diagnostics, e.g., the Movement Disorder Society (MDS) diagnostic guidelines for PD-MCI^1^ or the Diagnostic and Statistical Manual (DSM)^4^ diagnostic criteria for neurocognitive disorder, are mainly based on expert consensus rather than empirical findings. Remarkably, a recent scoping review on empirical findings in people with PD (PwPD) do not support either of those cognitive domain definitions which differ in how specific cognitive tests are assigned to cognitive domains^5^. This discordance exemplifies a broader challenge in neurodegenerative disorder diagnostics: the lack of empirically-derived, temporally-validated cognitive domain structures.

Existing empirical domain definitions of cognitive domains in PwPD rely almost exclusively on cross-sectional dimensionality reduction techniques such as factor analysis, which identify how cognitive test scores cluster at a single time point^5^. However, such static analyses may not accurately capture the temporal dynamics of cognitive change within and between cognitive domains. Longitudinal associations between cognitive test scores can diverge from cross-sectional patterns due to statistical phenomena such as Simpson’s paradox^6^, where group-level trends can obscure or reverse within-subject changes over time. Misinterpreting these dynamics risks undermining diagnostic accuracy and prognostic precision. Thus, understanding the longitudinal interplay between cognitive test scores is critical for refining diagnostic classifications and ensuring valid and reliable cognitive diagnostics in PD and other conditions. In addition, we can only reliably identify mechanisms of cognitive decline by linking cognitive-behavioral data with potentially relevant diagnostic and prognostic biomarkers, such as neuroimaging measures, genetic factors, or digital biomarkers, when based on valid and reliable cognitive diagnostics.

Understanding cognitive decline in neurodegeneration requires moving beyond static descriptions to examine temporal dynamics at two levels. First, we must identify the dynamic organization of cognition - how individual cognitive tests cluster together *over time* rather than at single timepoints, which may reveal fundamentally different structures. Second, we must characterize dynamic coupling between dimensions - how changes in one cognitive process accelerate, buffer, or reorganize changes in others. While previous longitudinal studies have explored cognitive trajectories in PD by focusing on predicting conversion to PD-MCI or PD dementia (PDD)^7–9^ or independent domain-specific declines^10^, few studies have examined how cognitive domains dynamically interact with one another longitudinally. Emerging network approaches such as panel graphical vector autoregression (GVAR) models^11^ enable direct examination of these temporal dependencies between cognitive dimensions.

This study addresses two research questions using longitudinal panel data from PwPD across the cognitive spectrum in the DEMPARK/LANDSCAPE study^12^. First, we aim to identify dynamic organization of cognitive test scores in PwPD in the DEMPARK/LANDSCAPE study and descriptively compare them to both a theoretically assumed domain structure^13^ and a previously derived empirical, cross-sectional domain structure^14^ from the same cohort. To adequately capture the complexity of cognitive functioning, we employ dynamic exploratory graph analysis (dynEGA)^15^, which performs a longitudinal clustering based on psychometric network models of cognitive test scores, indicating which test scores show similar changes over time. Second, we seek to characterize dynamic coupling, i.e., dynamics of cognitive change between identified cognitive dimensions to improve our understanding of cognitive decline mechanisms in PD and inform more precise diagnostic and therapeutic strategies. For this, we employ panel vector autoregression (GVAR) models^11^, enabling us to identify which dimensions predict other dimensions (or themselves) in the next measurement occasion.

## Results

### Sample descriptives

Our sample of PwPD from the DEMPARK/LANDSCAPE study (*N* = 355) consists of *n* = 185 individuals with Parkinson’s disease with cognitive functions within the demographically adjusted norms (normal cognition, PD-NC), *n* = 145 individuals with PD-MCI, and *n* = 25 individuals with PDD at baseline. Participants were *M* = 66.22 years old (SD = 7.76), 69.90% were male, and 31.10% were female. Descriptives of the sample characteristics of sociodemographic, clinical, and cognitive variables are provided in Table 1 and Tables S1–2.

**Table 1:** Descriptive characteristics of the sample at baseline (*N* = 355)

Variable	Value
Age in years	66.22 (7.76) [45–80]
Gender	
* Male*	245 (69.90%)
* Female*	110 (31.10%)
Years of education	13.73 (3.11) [8–20]
Disease duration in months	70.82 (57.27) [0–297]
Levodopa equivalent daily dose	667.37 (446.08) [22.20–2676.60]
UPDRS-III	20.61 (10.44) [1–55]
Hoehn & Yahr stages	
* 1*	69 (19.44%)
* 2*	192 (54.08%)
* 3*	78 (21.97%)
* 4*	14 (3.94%)
*unknown*	2 (0.56%)
Motor phenotype	
* Tremor dominant (TR-D)*	35 (9.86%)
* akinetic rigid (PIGD-D)*	279 (78.59%)
* Not determined (ND)*	31 (8.73%)
* Missing data*	10 (2.82E%)
GDS	3.07 (2.79) [0–14]
MMSE	28.34 (1.98) [18–30]
PANDA	22.91 (5.44) [3–30]
Cognitive status	
* PD-NC*	185 (52.11%)
* PD-MCI*	145 (40.85%)
* PDD*	25 (7.04%)

Data are mean (standard deviation) [range] or *n* (%) as appropriate. MMSE Mini-Mental State Examination (score range 0–30; cut-off for cognitive impairment ≤ 26); PANDA Parkinson Neuropsychometric Dementia Assessment (score range 0.30, cut-off for cognitive impairment ≤ 18); UPDRS-III Unified Parkinson’s Disease Rating Scale Part III (score range 0–132; cut-off for moderate motor severity ≥ 33; cut-off for severe motor severity ≥ 59); GDS Geriatric Depression Scale (score range 0–15; cut-off for mild depressive symptoms ≥ 6), PD-MCI Parkinson’s Disease normal cognition, PD-MCI Parkinson’s Disease mild cognitive impairment, PDD Parkinson’s Disease dementia.

### Dynamic organization of cognitive test scores

**Fig. 1:**
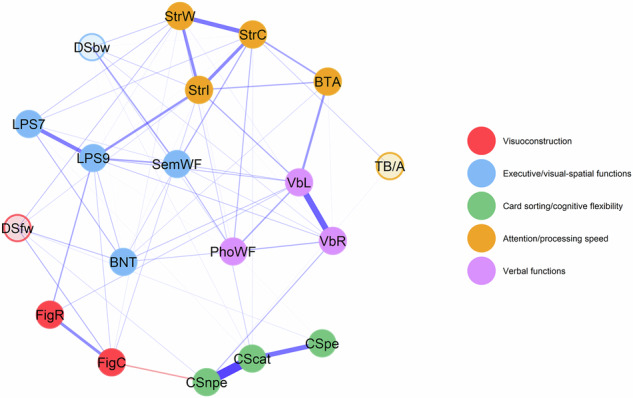
Network and dimensionality structure of cognitive test scores from people with Parkinson’s disease. Network and dimensionality structure of cognitive test scores from people with Parkinson's disease resulting from dynamic exploratory graph analysis. Blue edges indicate positive pairwise conditional associations; red edges indicate negative conditional associations. Node colors indicate assignment to dimensions as empirically derived by dynamic exploratory graph analysis. Transparent node color indicates network loadings below the cut-off for relevancy (.100). Dimension names were assigned by the authors based on mutual task features of cognitive test scores within dimensions. Abbreviations: BNT Boston Naming Test, BTA Brief Test of Attention, CScat Modified Card Sorting Test categories, CSnpe Modified Card Sorting Test non-perservative errors, CSpe Modified Card Sorting Test perservative errors, DSbw Digit Span backward, DSfw Digit Span forward, FigC Figures Copy, FigR Figures Recall, LPS7 Leistungsprüfsystem subtest 7, LPS9 Leistungsprüfsystem subtest 9, PhoWF phonematic Word Fluency, SemWF semantic Word Fluency, StrC Stroop color naming, StrI Stroop interference, StrW Stroop word reading, TB/A Trail Making Test quotient B/A, VbL Verbal Learning, VbR Verbal Recall.

We estimated a longitudinal psychometric network model of standardized cognitive test scores, representing how changes in each of the cognitive test scores relate to one another. The derived dimensionality structure indicates which scores showed similar rates of change over the course of four yearly assessments. Five temporally dynamic dimensions of cognitive test scores emerged from dynEGA based on the within-subjects rate of change over time (Fig. 1). Standardized network loadings, similar to factor loadings, for each test are presented in Table S3. The emergent dimensional structure diverged from the theoretically assumed cognitive domains (Table 1) and previously reported cross-sectional factor structures. The adjusted Rand index, a quantitative, descriptive measure for overlap of clustering solutions, indicated a value of 0.41 for the comparison of the dynEGA with the crossEGA structure and 0.21 compared to the theoretically assumed cognitive domain structure. These values indicate moderate agreement with the crossEGA structure and lower agreement with the theoretically assumed cognitive domain structure. In sum, the dynEGA revealed a reorganization of cognitive test scores compared to the static cognitive domain models.

Dimension 1 (*visuoconstruction*) encompasses two interdependent tasks, CERAD + ^16^ Figures Copy and Figures Recall. Both require cognitive processes such as spatial reasoning and motor praxis and share identical response formats (Tables S4–5). However, CERAD+ Figures Recall depends on stimuli encoded during the Figures Copy task, additionally requiring memory processes. The third task within dimension 1, Digit Span forward^17^, often described as a short-term memory task, showed very low network loadings, suggesting low reliability and potentially explaining the weak conceptual alignment with the other tasks.

Dimension 2 (*executive/visual-spatial functions*) consists of two tasks of the German test battery *Leistungsprüfsystem* (LPS)^18^ focusing on spatial rotation (LPS7) and spatial reasoning (LPS9), CERAD+ semantic word fluency, the CERAD+ Boston Naming Test (BNT), and Digit Span backward^17^. While most tasks in this dimension involve attentional components, they differ in specific cognitive processes and task features: LPS9 requires more executive processes (e.g., memory updating and planning) than LPS7 (both united by visual stimuli), while these executive processes are also reflected in semantic word fluency. Among others, the BNT requires semantic memory, which is a shared feature with LPS7 and semantic word fluency, and shares stimuli features with both LPS subtests.

Dimension 3 (*card sorting/cognitive flexibility*) consists of the Modified Card Sorting Test^19^ subscores, reflecting shared demands on logical reasoning, planning, set-shifting, memory updating, divided attention, and response inhibition.

Dimension 4 (*attention/processing speed*) includes the Stroop test^20^ subscores, the Brief Test of Attention (BTA)^21^, and the CERAD+ Trail Making Test (TMT) B/A quotient, though the latter showed very low network loadings. Common cognitive processes required are selective and sustained attention, semantic memory, and, partly, response inhibition. The timed response format of the Stroop subscores and the TMT reflect a processing speed component, which also applies to the rapid memory updating required in the BTA.

Dimension 5 (*verbal functions*) consists of the CERAD+ verbal learning and recall tasks alongside CERAD+ phonemic verbal fluency. Mutual cognitive processes required include semantic memory, response inhibition, and, depending on strategy use, vocabulary knowledge. These tasks also share a similar response types and modalities, suggesting verbal functions as the common component.

**Fig. 2:**
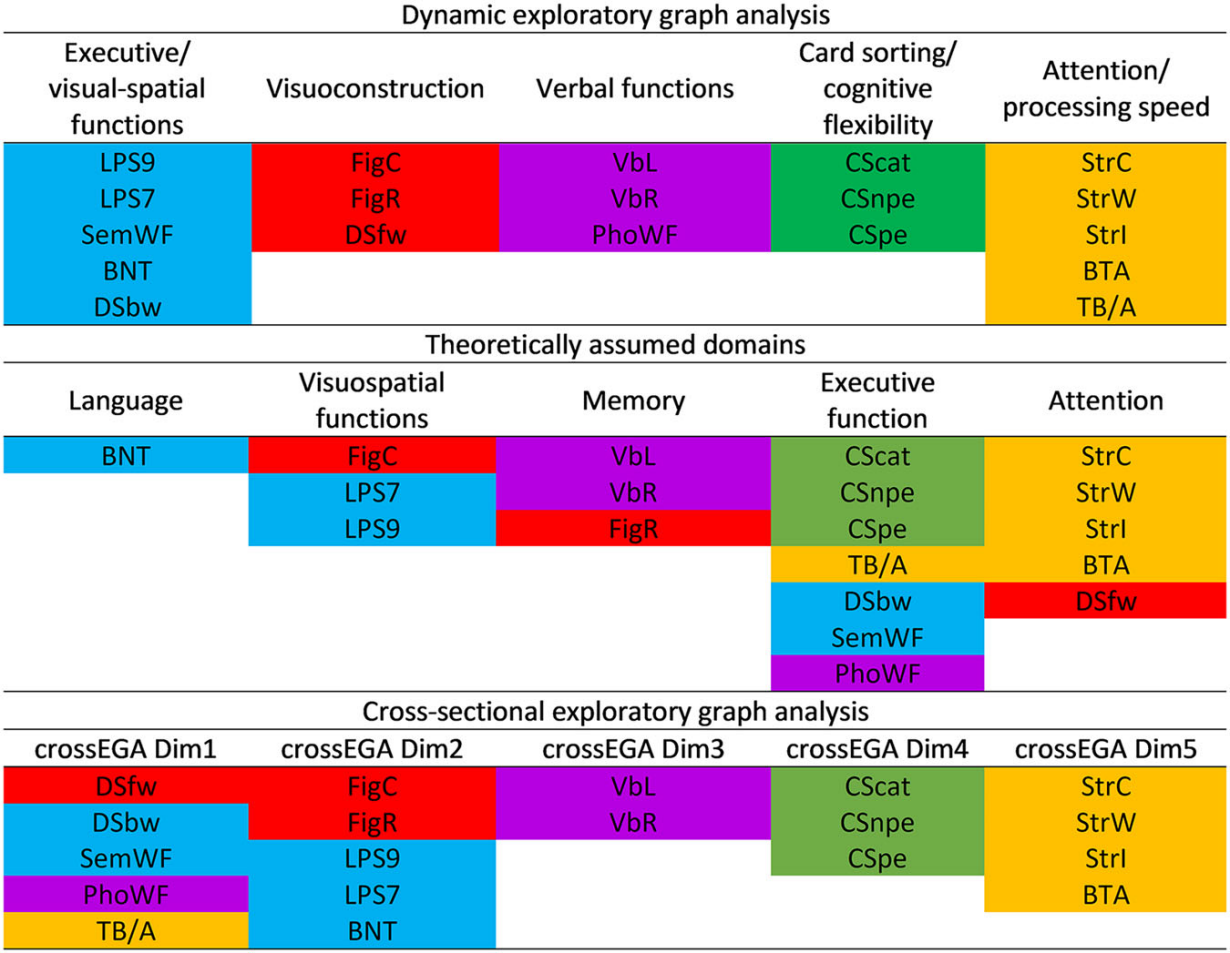
Comparison of cognitive domain structures. Cognitive domain structures of cognitive test scores from people with Parkinson's disease resulting from dynamic exploratory graph analysis, theoretical considerations, and cross-sectional exploratory graph analysis. Green edges indicate positive pairwise conditional associations; red edges indicate negative conditional associations. Node colors indicate assignment to dimensions as empirically derived by dynamic exploratory graph analysis. Abbreviations: BNT Boston Naming Test, BTA Brief Test of Attention, crossEGA cross-sectional exploratory graph analysis, CScat Modified Card Sorting Test categories, CSnpe Modified Card Sorting Test non-perseverative errors, CSpe Modified Card Sorting Test perservative errors, DSbw Digit Span backward, DSfw Digit Span forward, FigC Figures Copy, FigR Figures Recall, LPS7 Leistungsprüfsystem subtest 7, LPS9 Leistungsprüfsystem subtest 9, PhoWF phonematic Word Fluency, SemWF semantic Word Fluency, StrC Stroop color naming, StrI Stroop interference, StrW Stroop word reading, TB/A Trail Making Test quotient B/A, VbL Verbal Learning, VbR Verbal Recall.

In comparison to a previously published cross-sectional EGA model (crossEGA)^15^, only the *card sorting/cognitive flexibility* dimension remained stable across both models. All other dynEGA dimensions consist of test scores from two different crossEGA dimensions (Fig. 2), underscoring how cross-sectional structures may mislead the interpretation of the dynamic architecture of cognitive change in PD. In summary, dynEGA results showed different dimensionality patterns than the previously published crossEGA results.

Subsampling stability analyses showed that the dynEGA five-dimension solution could be replicated in 56.7% of subsamples, whereas a four-dimension solution appeared in 42.6%, and three- and six-dimension solution appeared in <1% of the iterations (Table S6). The average silhouette width of 0.69 indicated a good coherence of clustering during subsampling. A sensitivity analysis accounting for systematic effects of sex, age, education, motor impairment, and depression severity resulted in a comparable five-dimensional dynEGA solution. Both the subsampling and sensitivity analysis revealed overall stability of the original dynEGA solution; rather unstable test scores were already flagged with low network loadings (Digit Span forward, Digit Span backward, TMT B/A quotient) or cross-loadings (BTA) in the original dynEGA model. Another sensitivity analysis revealed no study center effects on the dynEGA structure. Detailed results of stability and sensitivity analyses can be found in Figs. S1–3.

When comparing dynEGA dimensions and theoretically assumed cognitive domain structures regarding their predictive utility for clinically relevant outcomes, linear mixed-effects models demonstrated a better model fit for dynEGA dimensions for predicting the Mini Mental State Examination (MMSE) total score (dynEGA: AIC = 3619.54, BIC = 3673.82, LL = −1798.77; theoretically assumed domains: AIC = 3621.87, BIC = 3676.16, LL = −1799.94; ΔAIC = 2.33). Detailed model information are reported in Tables S7–9.

### Dynamic coupling of identified cognitive dimensions

**Fig. 3:**
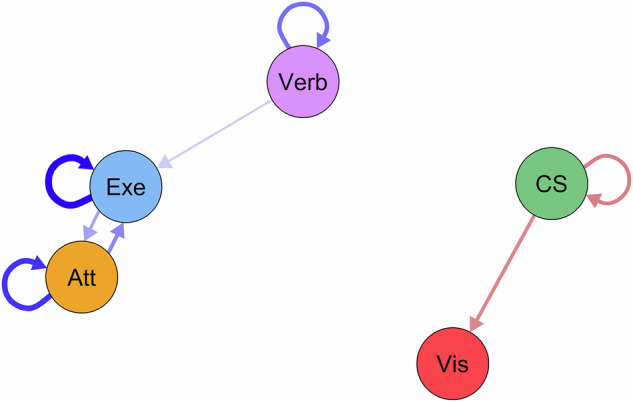
Temporal effects network model of cognitive dimensions. Temporal within-subject effect network model of cognitive dimensions derived by dynamic exploratory graph analysis. Blue edges indicate positive pairwise conditional associations; red edges indicate negative conditional associations. Node colors refer to dimensions identified by dynamic exploratory graph analysis as represented in Fig. 1). Abbreviations: Att dimension *attention/processing speed*, CS dimension *card sorting/cognitive flexibility*, Exe dimension *executive/visual-spatial functions*, Verb dimension *verbal functions*, Vis dimension *visuoconstruction*.

We performed panel GVAR analysis to evaluate if one dimension predicts another dimension (or itself) in the next measurement occasion. Panel GVAR analysis revealed dynamic coupling between the cognitive dimensions identified by the dynEGA (Fig. 3), revealing directional dependencies. These patterns suggest temporal ordering in cognitive decline rather than simultaneous deterioration across domains.

The temporal model showed positive autoregressions for dimensions *verbal functions*, *executive/visual-spatial functions*, and *attention/processing speed*: Individuals who perform well (or poorly) in one of these dimensions tend to maintain that relative standing at the next assessment. In contrast, *card sorting/cognitive flexibility* showed a negative autocorrelation, indicating that higher performance at one time point is associated with lower performance at the next assessment (or vice versa). This effect may indicate fluctuations in this dimension’s performance, showing as rebound pattern where deviations from an individual’s typical level are followed by corrections in the opposite direction.

Cross-dimensional couplings revealed temporal precedence relationships. *Executive/visual-spatial functions* and *attention/processing speed* show bidirectional positive coupling, indicating that performance in one of these dimensions positively predicts performance in the other dimension during subsequent assessment. *Verbal functions* positively predict performance in *executive/visual-spatial functions* in the next assessment. C*ard sorting/cognitive flexibility* shows a negatively directed edge towards *visuoconstruction*, indicating that better performance in *card sorting/cognitive flexibility* at one time point is coupled with worse performance in *visuoconstruction* at the next time point.

Sensitivity analysis in those with clinically meaningful cognitive impairment (PD-MCI, PDD) at baseline largely replicated this pattern but revealed two notable differences (see Fig. S4). First, positive coupling between dimensions *visual-spatial functions* and *verbal functions* reversed direction. Specifically, performance in *executive/visual-spatial functions* at one time point now predicts performance in *verbal functions* at the next time point of assessment. Second*, attention/processing speed* positively predicts *visuoconstruction*, an association not present in the temporal model of the total sample, potentially indicating that attentional resources become critical for maintaining visuospatial performance only after cognitive resource is depleted.

## Discussion

In this study, we assessed the temporal dynamic organization of cognitive dimensions exemplified in PwPD across varied cognitive statuses. We descriptively compared the longitudinal structure to a theoretically assumed cognitive domain structure^13^ and an empirical, cross-sectional cognitive domain structure^14^. We found that the longitudinal structure, revealing five cognitive dimensions, diverged substantially from both alternatives. Cognitive domains might not remain stable over time and static cognitive domain models may misrepresent dynamic cognitive organization. Analysis of dynamic coupling between cognitive dimensions revealed two distinct clusters of temporal effects, indicating domain-wise trade-offs in cognitive performance over time.

Longitudinal dynEGA dimensions, representing associated temporal change between cognitive test scores, i.e., clusters of test scores that showed similar patterns of change over time, differed from both the theoretically assumed cognitive domain structure^13^ and the empirical cross-sectional crossEGA dimensions^14^. This difference may be potentially explained by Simpson’s paradox, where trends observed at one level of analysis can be reversed when additional explanatory variables are included^6^. While full reversals of the statistical associations are unlikely in this context, the magnitude of associations between cognitive test scores may diminish when focusing on longitudinal within-subject effects rather than cross-sectional between-subject effects. Compared to the cross-sectional dimensions, bias introduced by method variance, i.e., the tendency that test scores correlate with each other because their instructions or stimuli are very similar to each other^22^, may be reduced in the longitudinal dimensions. While all test scores that are part of the same test paradigm (e.g., CERAD+ Figures Copy and Figures Recall) were consistently categorized into the same cross-sectional dimensions^14^, some pairs of test-scores were categorized into different dimensions (e.g., phonemic and semantic word fluency) in the longitudinal dimensions. The adjusted Rand index indicated a moderate overlap of clustering for the comparison of the dynEGA with the crossEGA structure and a lower overlap when compared to the theoretically assumed domain structure. While the adjusted Rand index is a quantitative, descriptive measure, a formal model comparison was not conducted due to cross-sectional versus longitudinal data structures, representing a limitation of the exploratory approach. In sum, differences of identified cognitive dimensions compared to cross-sectional cognitive domain structures may indicate mutual decline of specific cognitive processes required for successfully completing cognitive tasks (Table S4), reflecting a deeper latent structure than cross-sectional task similarity alone.

The dissociation between cross-sectional (static) and longitudinal (dynamic) cognitive organization has important conceptual implications. First, our results challenge the assumption of temporally stable cognitive domains. Rather, they indicate that correlations between cognitive test scores in cross-sectional data do not necessarily reflect how cognitive processes required in cognitive tasks mutually decline with each other longitudinally. Static cognitive domain models thus may misrepresent dynamic cognitive organization in PwPD – potentially explaining the highly heterogeneous findings of studies aiming to identify a cognitive domain structure from empirical data^5^ – whereas longitudinal clustering identified by dynEGA captures the co-trajectory of cognitive test score change, representing a sequential or staged proregression of different cognitive functions. Second, our findings highlight the need for consistent and empirically based diagnostic criteria for valid and reliable cognitive subtype diagnostics in both clinical practice and research. Such cognitive subtype diagnoses are not only prognostic for clinical disease progression^2^, but in research are also often linked to potentially relevant diagnostic and prognostic biomarkers, such as neuroimaging measures^23^, genetic factors^24^, or digital biomarkers (e.g., actigraphy data)^25^. Considering dynamic cognitive organization may increase the validity and reliability of cognitive subtyping based on cognitive domains and, in turn, increase the quality and validity of findings linking cognitive phenotyping to other data modalities for uncovering mechanisms and risk factors of cognitive decline. Our findings showed that longitudinal dynEGA dimensions performed better than the theoretically assumed domains in modeling the MMSE total score as an independent, global cognitive outcome, demonstrating the potential of improving prognostic accuracy of clinically relevant variables. Future studies should expand these findings by evaluating the predictive utility of different cognitive functioning structures for cognitive outcomes and dementia conversion in independent samples. Further, they should investigate the potential predictive utility for other relevant and prognostic biomarkers as described above. Third, our findings may have profound clinical implications. Cognitive screening tests, such as the widely-used MMSE, usually result in a global cognition total score but also rely on clustering of cognitive subitems. A recent study has utilized categorizations of single cognitive screening tasks to cognitive domains in another cognitive screening test, the Montreal Cognitive Assessment, to create proxies for standardized scores of conventional neuropsychological tests^26^. Showing the increased predictive utility of longitudinal dynEGA dimensions for modeling the MMSE total score suggests that considering temporal information on classifications of cognitive test scores into domains in cognitive screening tests may also help to improve diagnostic accuracy of such screenings tests that are of high clinical relevance for detecting cognitive impairment in PwPD. In addition, cognitive subtyping may be used for planning appropriate personalized cognitive training interventions^3,27^ and could also benefit from taking temporal information about the cognitive reorganization into account. In sum, our findings call into question the reliability and validity of widespread guidelines for cognitive diagnostics based on cognitive domains, such as the MDS guidelines for assessment of PD-MCI^1^ or the DSM criteria for neurocognitive disorder^4^.

Similar to factor loadings in factor analyses, it is possible to compute network loadings for dynEGA dimensions^15^. Some variables (Digit Span forward, Digit Span backward, TMT quotient B/A) showed very low network loadings, indicating low association of these scores with dynEGA dimensions. Potential reasons might include a generally low reliability of such scores, floor/ceiling effects, or unique longitudinal change patterns that could not be captured by the psychometric network analysis underlying dynEGA, e.g., non-linear trajectories. The TMT quotient already showed very low network loadings in the previously published crossEGA^14^, potentially reflecting issues with floor effects resulting from coding failed TMT part B attempts as maximum time. However, visual inspection of the test score distribution (Fig. S5) does not reveal any ceiling/floor effects for our study. Nonetheless, variables with low network loadings should not automatically be discarded as non-informative in clinical practice. For example, Digit Span forward and Digit Span backward showed low network loadings for the dynEGA dimensions, but the highest network loadings for dimension 1 reported in the crossEGA results^14^. For cross-sectional purposes of cognitive diagnostics, the Digit Span test scores might still be informative measures to assess cognitive performance in PwPD. Future research should thus evaluate the significance of network loadings, from both dynEGA and crossEGA, about the informativeness of cognitive test scores and its implications for the compilation of neuropsychological test batteries.

Although we are not aware of existing similar analyses in the context of Alzheimer’s disease (AD) or other clinical samples, it seems likely that the described temporal reorganization is a general mechanism and not restricted to cognitive functioning in PwPD. As different clinical samples (e.g., PD vs. AD) show different cross-sectional cognitive profiles^28^, they might also show differences in their specific configurations of dynamic reorganization. In fact, dynamic reorganizations might be seen as the defining measurable expressions of different (neurodegenerative) disease mechanisms affecting cognition. Thus, future studies should investigate the temporal reorganization of cognitive domains in other clinical samples such as AD.

Panel GVAR analysis revealed dynamic coupling of cognitive dimensions, indicating if one dimension predicts another dimension (or itself) in the next measurement occasion. Notably, *card-sorting/cognitive flexibility* shows a directed negative association towards *visuoconstruction*. This finding aligns remarkably with the dual-syndrome hypothesis of cognitive impairment in PD^29,30^, which postulates two distinct but overlapping paths of cognitive decline in PD: (1) an early-onset fronto-striatal impairment, manifesting as dopamine-modulated deficits in executive functions and attention, and (2) a later-onset cholinergic-modulated posterior and temporal lobe impairments showing as language (semantic fluency), memory, and visual-spatial dysfunctions, with more rapid decline to PDD. The negative temporal association between *card sorting/cognitive flexibility* and *visuoconstruction* could reflect the separation of early executive deficits of the fronto-striatal path from later visual-spatial deficits of the posterior-cortical path postulated by the dual-syndrome hypothesis^30^. Importantly, there is no negative association from *card sorting/cognitive flexibility* towards the *executive/visual-spatial dimension*, as the *executive/visual-spatial* dimension seems to reflect both fronto-striatal and posterior-cortical functions, evidenced by (a) the strong attentional and partly executive components when analyzing the underlying cognitive processes, stimuli, and response characteristics (Table S4), and (b) cross-loadings of LPS9 with the *attention/processing speed* dimension. Thus, the *visuoconstruction* dimension seems to reflect the posterior-cortical impairments better than ambiguous visual-spatial tasks additionally relying on executive processes.

Similar to findings of dynamic organization of cognitive domains, dynamic couplings may potentially reflect disease-specific mechanisms and might be related to the dual-syndrome hypothesis of PD. However, without an age-matched control sample, it remains unclear whether the observed temporal dynamics reflect PD-specific processes or general aging-related processes. Future studies should therefore investigate this analytical framework in both healthy and other clinical samples such as AD. In sum, findings of dynamic couplings between cognitive dimensions may be related to theories about the longitudinal development of cognitive functioning in aging or neurodegenerative diseases such as the dual-syndrome hypothesis in PD.

One limitation of the present analyses is that some variables showed potential ceiling effects. However, visual inspection showed distributional shifts decreasing ceiling effects over time (Supplementary Fig. S5), indicating that the longitudinal approach of our analyses could compensate for this potential bias. Further, including all test scores regardless of potential ceiling effects increased comparability with the theoretical cognitive domain structure^13^ and the cross-sectional dimensional structure^14^ from the same cohort. In addition, our sample is potentially subject to Berkson’s bias, a sampling bias that occurs when sample selection is based on clinical variables that are later included in the analyses^31^. For our analyses, we included data from all participants for whom cognitive test data were available at the fourth time point of the DEMPARK/LANDSCAPE study. Hence, participants who dropped out of the study during the first three time points of assessments due to severe cognitive or motor symptoms are not included in this sample. Consequently, our findings should only be generalized with caution to the general population of PwPD and especially to the subgroup of PwPD with severe cognitive or motor impairments. To address this potential issue and to focus on mechanisms of cognitive decline, we performed additional GVAR analyses with a subsample of people who already showed cognitive impairment at baseline assessment. Results of these analyses mostly confirmed our findings from the total sample. Further, the majority of our sample showed an akinetic-rigid motor phenotype exhibiting postural instability and gait difficulty (PIGD-D), which has been associated with greater cognitive impairment and decline compared to the tremor dominant (TR-D) and not determined (ND) motor phenotype^32,33^. Hence, generalizability of the findings to other motor phenotypes may be limited and should be investigated in future studies.

Despite potential confounders including sex, education, depressive symptoms, and motor symptoms, our findings demonstrated overall stability across sensitivity analyses and subsampling procedures. Minor variations in dimensional structure were explained by low network loadings and cross-loadings of specific variables. However, the influence of individual variables on cognitive clustering warrants further investigation, for instance, whether cognitive clustering differs between males and females. Results remained stable when accounting for study center effects in sensitivity analyses. Future studies should also investigate how learning and practice effects, i.e., improvements in mean test scores due to repeated measurement, influence covariance structures, to determine whether and how researchers should account for such effects before applying dynEGA or related techniques.

Further, the effects of therapeutic interventions on the temporal dynamics could not be accounted for in the present analyses due to missingness of this information in the data set. Therefore, temporal dynamics could partly reflect intervention effects rather than intrinsic cognitive organization. First, the levodopa equivalent daily dose (LEDD was only available for baseline assessment but not for later assessments. Dopaminergic medication could have influenced the underlying covariance matrix as differential effects are reported on various cognitive outcomes. For example, positive effects have been reported on card sorting tasks, task switching or working memory tasks, whereas negative effects have been reported on learning or gambling and decision making^29^. Due to its mixed and non-linear effects on cognitive functions, identifying, quantifying, and adjusting for medication effects when applying psychometric network analysis such as the dynEGA is complex and requires further investigation. Second, the data set does not contain information on deep brain stimulation (DBS). As DBS was not specified as an exclusion criterion in the DEMPARK/LANDSCAPE study (neither at baseline nor during the course of data collection), some participants may have received DBS before or during the study period. This may have impacted the covariance structure of cognitive test scores due to DBS’ differential effects on cognitive functions, including reported negative effects on performance in long-term memory and verbal fluency tasks^34^. In sum, despite the potential confounders discussed, our findings did not seem to be meaningfully biased or unstable, as indicated by the results of sensitivity analyses and subsampling procedures.

Our results of the GVAR analyses mainly focused on the temporal within-subject network model. However, because yearly measurement intervals may underrepresent measures capturing meaningful short-term changes happening faster than can be represented by first-order derivates across four consecutive observations, the contemporaneous fixed-effect within-subject network model should be taken into account (Fig. S4A). This model identifies bidirectional predictions between variables within the same measurement occasion after accounting for temporal information, capturing effects occurring faster than the measurement intervals^35^. The temporal and contemporaneous models show similar patterns, though the contemporaneous model shows an additional positive edge between verbal functions and *card sorting/cognitive flexibility* and does not include the negative edge between *card sorting/cognitive flexibility and visuoconstruction*. Future research should incorporate more frequent measurement occasions to better capture short-term cognitive dynamics to reliably estimate longitudinal within-subject network models.

Generalization of our findings is limited by heterogeneity in cognitive test batteries across studies^5^ which may identify different temporal dimensions. For instance, the cognitive assessment battery administered in this study does not fully conform to the recommendations for cognitive assessments in PD-MCI^1^ as it includes only one task assessing verbal functions according to the MDS guidelines instead of two. Consequently, both cognitive status classification and the dimensional structure may differ from studies using MDS level II compliant batteries. Importantly, we do not claim to have identified the “true” structure of cognitive domains. Although the longitudinal dynEGA dimensions showed better predictive utility in comparison to the theoretically assumed domains, we do not recommend to simply replace one categorical structure by another. Rather, our findings highlight the importance of empirical studies on cognitive domain organization and the added value of longitudinal approaches to consider dynamic reorganization. Instead, future research should explore new measures of cognitive performance based on the concept of cognition as a complex, interconnected network, moving beyond categorical concepts such as cognitive domains that neglect both temporal dynamics and task-related variances.

The present study shows several strengths. Employing psychometric network analysis allowed us to analyze and visualize cognitive functioning as an interconnected, complex system rather than discrete cognitive domains. Additionally, the longitudinal approach enabled us to identify the temporal dynamics within the network of cognitive functions. Identifying these temporal dynamics may contribute to increase diagnostic accuracy and prognostic precision in cognitive diagnostics by questioning the assumption of temporally stable cognitive domains, possibly initiating refinements in how cognitive tests are assigned to cognitive domains based on longitudinal empirical findings and a stronger focus on specific task features. Another strength is that we were able to descriptively compare various cognitive clustering structures (a theoretical structure^13^, the cross-sectional dimensional structure^14^, and the longitudinal dimensional structure) all relying on the same cognitive test battery in the same cohort of PwPD. This increased comparability of these different approaches, which is usually limited by a great variety of the cognitive test scores included in the analyses^5^, and, consequently, validity of the found differences.

To conclude, our findings provide evidence for a dynamic reorganization of cognitive domains in PwPD, challenging the foundational assumption of temporally stable cognitive domains and indicating that static cognitive domain models may misrepresent dynamic cognitive organization. This challenges the validity and reliability of cognitive diagnoses relying on the concept of cognitive domains, such as diagnoses based on the criteria for PD-MCI^1^ or neurocognitive disorder^4^. The network perspective on cognitive functioning presents as a useful approach to complement diagnostic guidelines by creating awareness of the interdependence of cognitive test scores and the definitional issues that come with the traditional classification system of cognitive test scores. Identifying the dynamic coupling between cognitive dimensions allows to refine predictions from disease-specific theories of cognitive decline, such as the DSH in PD^30^ in this study, and to derive new concepts within this framework for other clinical samples. To conclude, our study established a framework for reconceptualizing the (re)organization and temporal coupling of cognitive dimensions.

## Methods

### Participants

We analyzed longitudinal data from the first four annual assessment time points (i.e., up to the 36-months follow-up) of the DEMPARK/LANDSCAPE study^12^, a completed, observational, prospective multi-center cohort study that aimed to characterize the natural course of cognitive decline in PwPD. To ensure appropriate sample size for analyses, we did not include data after the 36-months follow-up. Participants were consecutively recruited from nine movement disorder centers across Germany, entering the study at different time points relative to their diagnosis. Inclusion criteria were age between 45 and 80 years and a diagnosis of idiopathic PD based on the United Kingdom Parkinson’s Disease Society Brain Bank criteria. The total sample included individuals with different cognitive statuses: PwPD with cognitive functions within the demographically adjusted norms (normal cognition; PD-NC), PD-MCI, and PDD. Cognitive diagnoses were based on the diagnostic criteria for cognitive impairment that were available at the time of study set-up^36,37^. To distinguish PDD from dementia with Lewy bodies, PDD was assumed if parkinsonism preceded dementia by more than 12 months, whereas DLB was assumed if dementia occurred before or concurrently, in accordance with current diagnostic guidelines at the time of recruitment^12,38^. From the original sample (*N* = 711), we excluded those without available data for the fourth assessment time point (*n* = 356). The final data consisted of 24,372 valid data points (90.3%) across 19 cognitive test scores of *N* = 355 individuals with PD with different cognitive statuses and four time points. Higher proportions of missing data were observed for time points after baseline and for the Modified card sorting items, presumably due to task complexity. Detailed information on missing data is provided in Tables S10–13.

All procedures complied with the ethical standards of the relevant national and institutional committees on human experimentation and with the Helsinki Declaration of 1975, as revised in 2008. Study procedures of the DEMPARK/LANDSCAPE project were approved by the Ethics Committee of Philipps University Marburg (approval-no. 178/07) in March 2009, and thereupon by the ethics committees of the participating centers. All participants gave written informed consent prior to their inclusion in the study.

### Cognitive and clinical assessment

Cognitive functioning was assessed using a comprehensive cognitive test battery administered as part of the DEMPARK/LANDSCAPE study. Table 2 provides an overview of the cognitive tests and their classification into theoretically assumed cognitive domains, based on expert consensus^13^. Tables S4–5 provide details on cognitive task features, such as type of cognitive process, stimuli and responses. All neuropsychological assessments were administered in a fixed order. Assessments were administered by various trained examiners both across and within study centers. After standardizing and demographically adjusting the raw cognitive test scores using published normative data to percentiles, z-scores, or T-scores, we uniformly transformed all standardized scores into z-scores. Tables S14–17 and Fig. S5 present descriptive statistics and score distributions for all cognitive test scores. The data also include sociodemographic variables (age, sex, years of education), clinical variables (disease duration; levodopa equivalent daily dose; Unified Parkinson’s Disease Rating Scale Part III, UPDRS-III^39^; Hoehn & Yahr stage^40^; PD motor phenotype; Geriatric Depression Scale, GDS^41^), and cognitive variables for the global cognitive status (Mini-Mental State Examination^42^; Parkinson Neuropsychometric Dementia Assessment^43^; cognitive status based on clinical evaluation). PD motor phenotypes (tremor-dominant, TR-D; akinetic-rigid exhibiting postural instability and gait difficulty, PIGD-D; not-determined, ND) were based on UPDRS-III scores according to the procedure that was previously reported in other DEMPARK/LANDSCAPE analyses^32,33^. All assessments were conducted while participants were in the ON medication state.

**Table 2:** Cognitive tests and corresponding theoretically assumed cognitive domains in the DEMPARK/LANDSCAPE study

Theoretically assumed cognitive domain	Test score	Abbreviation
Attention	Digit Span forward^17^	DSfw
	Brief Test of Attention^21^	BTA
	Stroop word reading^20^	StrW
	Stroop color naming^20^	StrC
	Stroop interference^20^	StrI
Executive functions	CERAD+ Semantic Word Fluency^a^	SemWF
	CERAD+ Phonematic Word Fluency^a^	PhoWF
	CERAD+ Trail Making Test B/A^a^	TBA
	Digit Span backwards^17^	DSbw
	Modified Card Sorting Test categories^19^	CScat
	Modified Card Sorting Test non-perservative errors^19^	CSnpe
	Modified Card Sorting Test perservative errors^19^	CSpe
Memory	CERAD+ Figures Recall^a^	FigR
	CERAD+ Verbal Learning^a^	VbL
	CERAD+ Verbal Recall^a^	VbR
Language	CERAD+ Boston Naming Test^a^	BNT
Visual-spatial functions	CERAD+ Figures Copy^a^	FigC
	Leistungsprüfsystem subtest 7 Spatial Rotation^18^	LPS7
	Leistungsprüfsystem subtest 9 Spatial Reasoning^18^	LPS9

^a^Part of the extended Consortium to Establish a Registry for Alzheimer’s disease neuropsychological test battery (CERAD + )^16^.

### Statistical analysis

We performed all statistical analyses using *R* version 4.3.1^44^. We provide the code used for statistical analyses as Supporting Information B.

First, we tested all cognitive test variables on the assumption of stationarity in the data using *R* package *tseries*^45^. Because the Kwiatkowski-Phillips-Schmidt-Shin (KPSS) test indicated stationarity for all variables except for CERAD+ Verbal Learning (*p* = 0.032), we did not perform any additional measures regarding systematic trends.

To identify dimensions of cognitive test scores that co-evolve longitudinally, addressing our first research question, we applied dynEGA^15^ to the full sample across all four timepoints. Unlike traditional factor analysis, which aims to identify cross-sectional latent factors, dynEGA is a clustering method that detects clusters of variables showing associated temporal change within individuals. We applied dynEGA with the Louvain algorithm to a glasso-regularized gaussian graphical model using *R* package *EGAnet*^46^. The dynEGA used four consecutive observations (*n.embed* = *4*) to estimate first-order derivatives (*use.derivates* = *1*) of the variables, which can be interpreted as velocity or rate of change over time within individuals^46^. Missing data were handled via pairwise deletion by the dynEGA function, i.e., correlations were computed using all available cases for each variable pair. Dimensions identified by dynEGA represent clusters of cognitive test scores that changed with each other over time within individuals. The Louvain algorithm uses the community clustering method^47^, applying a community detection algorithm repeatedly to the same network and selecting the modal community solution across all iterations (*N* = 1000). We then computed standardized network loadings, which are conceptually analogous to factor loadings, but have different cut-offs for effect size interpretation: values of 0.15 are considered small, 0.25 moderate, and 0.35 large^48^. For a descriptive, quantitative comparison between the dynEGA, the crossEGA, and the theoretically assumed cognitive domain structure, we computed the adjusted Rand index using R package *mclust*^49^. The index quantifies the similarity between cluster solutions by assessing the extent to which variable pairs have been assigned to the same or to different clusters across solutions^50^. Possible values range from −1 to 1 where 1 indicates perfect agreement between clustering solutions, 0 indicates agreement at chance level, and negative values indicate agreement worse than expected by chance.

To assess the stability of the identified dynEGA solution, we performed subsampling procedures with *N* = 1000 iterations and subsamples consisting of 85% of the original sample. Based on the frequencies of variable pairs clustering together, we computed the average silhouette width, which quantifies how stable the groups of variables are that tend to appear together^51^. Values above 0.50 are acceptable, values from 0.70 indicate strong structures with stable clustering^51^. To account for potential confounders, we regressed sex, age, years of total education, PD-related motor impairment (UPDRS-III score), and depression severity (GDS score) on each of the cognitive test scores and then calculated the residualized cognitive test scores as the difference between the observed cognitive test scores and the scores predicted by the regression models. We then performed dynEGA on these residualized scores. Another sensitivity analysis accounted for study center effects by linearly regressing a one-hot encoded study center variable on the cognitive test scores.

To compare the predictive utility of dynEGA dimensions versus the theoretically assumed cognitive domain structure, we performed linear mixed-effect models with the MMSE total score as dependent variable, using the *lmer* function of *R* package *lme4*^52^. Predictors were time and (a) the unweighted mean of z-scores of cognitive tests included in each dynEGA dimensions that was computed for each person and time point and (b) the unweighted mean of z-scores of cognitive tests according to the theoretically assumed domains that was computed for each person and time point. Time and the dynEGA dimension mean scores or theoretical cognitive domain mean scores were fixed effects; a random intercept was specified for each individual. We compared the dynEGA model and theoretical cognitive domain model using the Akaike information criterion (AIC), Bayesian information criterion (BIC), and log-likelihood (LL).

To examine how cognitive dimensions are dynamically associated with one another over time, addressing our second research questions, we computed dimension-weighted means for each participant of the total sample based on the network loadings, resulting in five scores per participant, one for each dynEGA dimension. Because dynEGA identifies clusters of cognitive tests that share similar temporal dynamics, these dimension-weighted means can be interpreted as a cognitive dimension composite score reflecting the temporal coherence of tests within dynamic cognitive dimensions. Figure S6 shows descriptive statistics of these scores for each time point. The KPSS test indicated stationarity for all dimension-weighted composite scores used in the analyses. The scores were then used as input for a Graphical Vector Autoregression (GVAR) model using the *R* package *psychonetrics*^53^. The time variable was defined as the number of months since baseline (0, 12, 24, 36). We report a temporal within-subject network model indicating whether one dimension predicts another dimension (or itself) at the next measurement occasion. This temporal model was pruned (*α* = 0.05) non-recursively using R-package *psychonetrics*^53^, applying the Hochberg correction for multiple corrections. The saturated model is reported in Fig. S7. A contemporaneous within-subject effect network model, indicating if two variables predict one another within the same measurement occasion after taking temporal information into account, and a between-person network model are reported in Fig. S8.

To address a potential selection bias in our study favoring inclusion of PwPD that stay cognitively and bodily healthy over time, interfering with research question two focusing on mechanisms of cognitive decline, we computed another exploratory GVAR model that includes only PwPD already showing cognitive impairment at baseline as sensitivity analysis.

## Supplementary information

Supplementary information

Supplementary information B: Analysis Code

## Data Availability

The data supporting this study’s findings are available on reasonable request from the corresponding author Elke Kalbe in consultation with the DEMPARK/LANDSCAPE consortium. The data are not publicly available due to privacy and ethical restrictions. The underlying statistical code for this study is available as Supplementary Material B and can be accessed via the online version of the article.

## Code availability

The underlying statistical code for this study is available as Supplementary Material B and can be accessed via the online version of the article.
