# Supplementary material for: Temporal dynamics of cognitive functioning in people with Parkinson’s disease
Source: NPJ Parkinsons Dis. 2026 Apr 6;12:86. doi: 10.1038/s41531-026-01338-3 (PMC13057304; doi:10.1038/s41531-026-01338-3)
Supplement: Supplementary file 1 — Supplementary information [file 41531_2026_1338_MOESM1_ESM.docx]

**SUPPORTING INFORMATION A**

**This file contains Supplementary Material to**

Scharfenberg, D., Kalbe, E., Ophey, A., Balzer-Geldsetzer, M., Berg, D., Hilker-Roggendorf, R., Kassubek, J., Liepelt-Scarfone, I., Mollenhauer, B., Reetz, K., Riedel, O., Roeske, S., Schulz, J. B., Storch, A., Trenkwalder, C., Witt, K., Dodel, R., & Deserno, M.K. (Manuscript submitted for publication in *npj Parkinson’s disease*). *Temporal dynamics of cognitive functioning in people with Parkinson’s disease.*

**Corresponding author**s:

Daniel Scharfenberg

Medical Psychology | Neuropsychology and Gender Studies, Center for Neuropsychological Diagnostics and Intervention (CeNDI), University Hospital Cologne and Faculty of Medicine, University of Cologne, Cologne, Germany

Kerpener Str. 62, 50937 Köln

Phone: +49 221 478-86485

daniel.scharfenberg@uk-koeln.de

Prof. Elke Kalbe

Medical Psychology | Neuropsychology and Gender Studies, Center for Neuropsychological Diagnostics and Intervention (CeNDI), University Hospital Cologne and Faculty of Medicine, University of Cologne, Cologne, Germany

Kerpener Str. 62, 50937 Köln

Phone: +49 221 478-6669

elke.kalbe@uk-koeln.de

**Table of Contents**

| **Material** | **Description** | **Page** |
| --- | --- | --- |
| *Supplementary Figures* |  |  |
| Figure S1 | Subsampling cluster heat-map | 1 |
| Figure S2 | dynEGA sensitivity analysis (demographic & clinical variables) | 2 |
| Figure S3 | dynEGA sensitivity analysis (study center) | 3 |
| Figure S4 | GVAR sensitivity analysis with impaired subsample | 4 |
| Figure S5 | Cognitive test score distributions | 5 |
| Figure S6 | Longitudinal plot of dimension-weighted means | 6 |
| Figure S7 | Saturated (unpruned) GVAR model | 7 |
| Figure S8 | Panel GVAR contemporaneous and between-subject models | 8 |
| *Supplementary Tables* |  |  |
| Table S1 | Descriptive baseline characteristics of cognitive status groups | 9 |
| Table S2 | Descriptive baseline characteristics of PD motor phenotypes | 11 |
| Table S3 | dynEGA network loadings | 13 |
| Table S4 | Task features of cognitive test scores | 14 |
| Table S5 | Task feature definitions | 16 |
| Table S6 | dynEGA subsampling dimension results | 17 |
| Table S7 | Model fit comparison MMSE score | 18 |
| Table S8 | Model summary dynEGA MMSE score | 18 |
| Table S9 | Model summary theoretical domains MMSE score | 19 |
| Table S10 | Missing/valid pairwise-observations t0 | 20 |
| Table S11 | Missing/valid pairwise-observations t1 | 22 |
| Table S12 | Missing/valid pairwise-observations t2 | 24 |
| Table S13 | Missing/valid pairwise-observations t3 | 26 |
| Table S14 | Descriptive statistics of cognitive test scores (full sample) | 28 |
| Table S15 | Descriptives of cognitive test scores (PD-NC) | 29 |
| Table S16 | Descriptives of cognitive test scores (PD-MCI) | 30 |
| Table S17 | Descriptives of cognitive test scores (PD-D) | 31 |

**SUPPLEMENTARY FIGURES**

**Fig. S1**


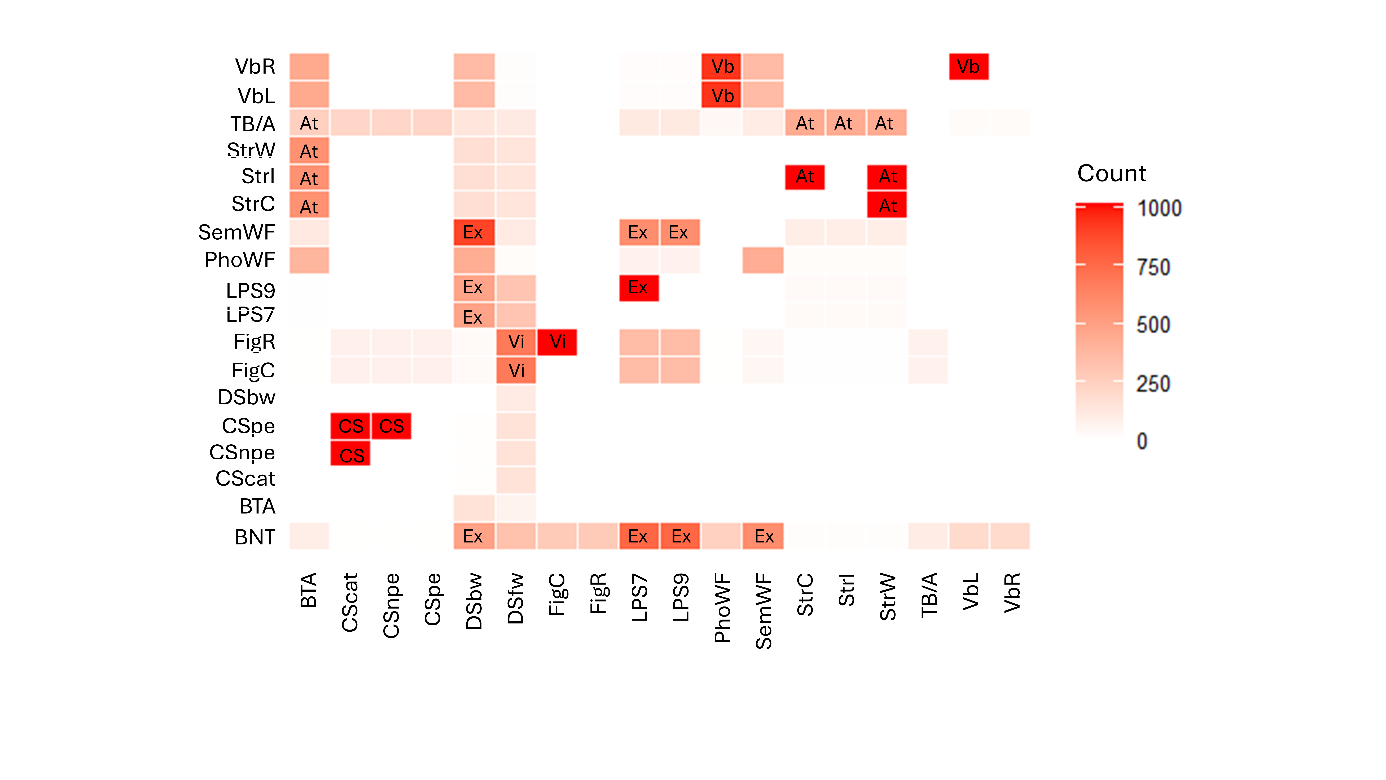


Heatmap depicting frequencies of variable pairs clustered together during subsampling procedures with N = 1000 iterations of 85% of the original sample. Abbreviations within cells refer to affiliation with dimensions identified in the original dynamic exploratory graph analysis.
Abbreviations: At, dimension *attention/processing speed*; BNT, Boston Naming Test; BTA, Brief Test of Attention; CS, dimension *card sorting/cognitive flexibility*; CScat, Modified Card Sorting Test categories; CSnpe, Modified Card Sorting Test non-perservative errors; CSpe, Modified Card Sorting Test perservative errors; DSbw, Digit Span backwards; DSfw, Digit Span forward; Ex, dimension *executive/visual-spatial functions*; FigC, Figures Copy; FigR, Figures Recall; LPS7, Leistungsprüfsystem 7; LPS9, Leistungsprüfsystem 9; PhoWF, phonematic Word Fluency; SemWF, semantic Word Fluency; StrC, Stroop color naming; StrI, Stroop interference; StrW, Stroop word reading; TB/A, Trail Making Test B/A; Vb, dimension *verbal functions*; VbL, Verbal Learning; VbR, Verbal Recall; Vi, dimension *visuoconstruction*.

**Fig. S2**


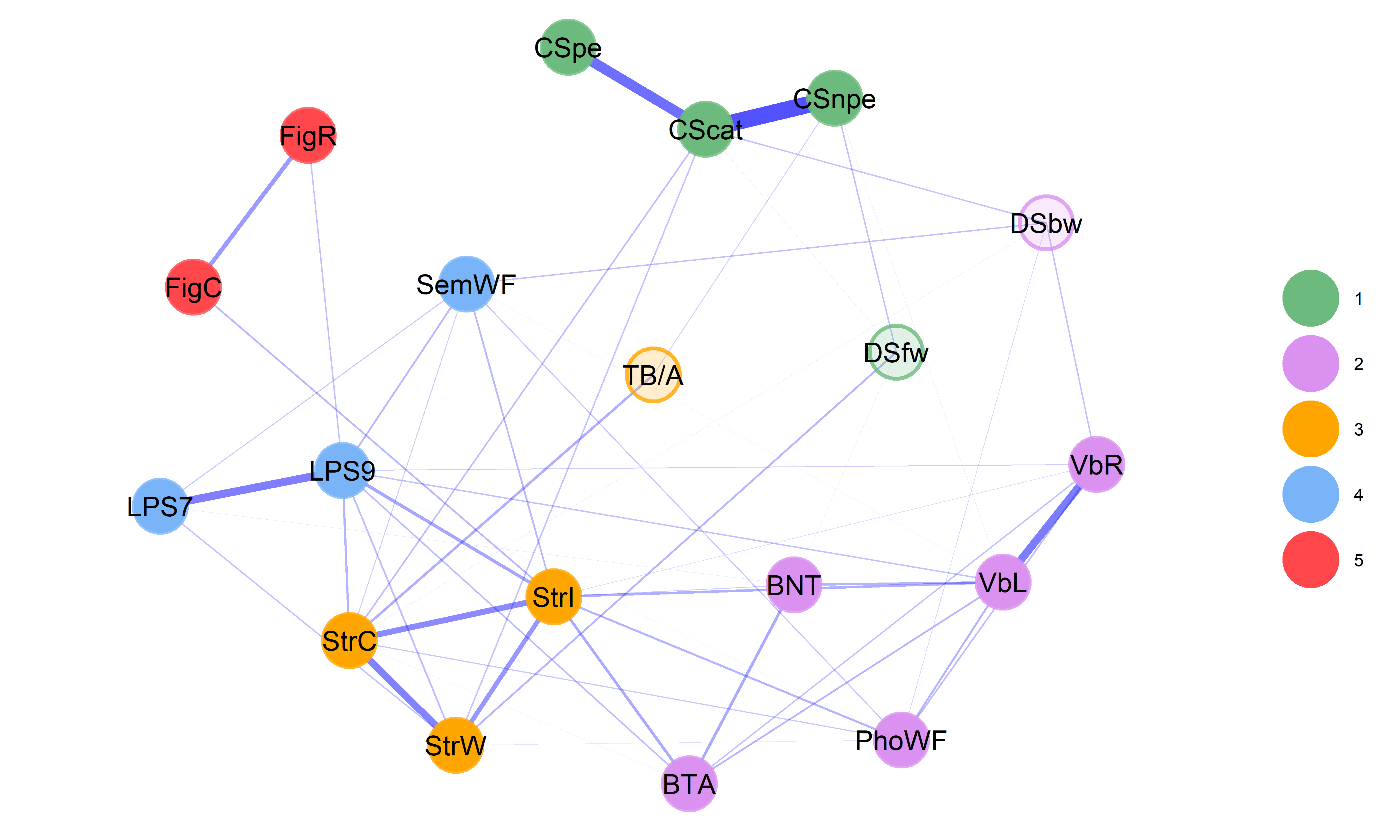


Network and dimensionality structure of residualized cognitive test scores (controlled for sex, age, years of total education, PD-related motor impairment, and depression severity) in individuals with PD resulting from dynamic exploratory graph analysis. Blue edges indicate positive pairwise conditional associations; red edges indicate negative conditional associations. Node colors indicate assignment to dimensions as empirically derived by dynamic exploratory graph analysis. Transparent node color indicates network loadings below the cut-off for relevancy (.100) in the original dynEGA solution. Abbreviations: BNT, Boston Naming Test; BTA, Brief Test of Attention; CScat, Modified Card Sorting Test categories; CSnpe, Modified Card Sorting Test non-perservative errors; CSpe, Modified Card Sorting Test perservative errors; DSbw, Digit Span backwards; DSfw, Digit Span forward; EGA, exploratory graph analysis; FigC, Figures Copy; FigR, Figures Recall; LPS7, Leistungsprüfsystem 7; LPS9, Leistungsprüfsystem 9; PhoWF, phonematic Word Fluency; SemWF, semantic Word Fluency; StrC, Stroop color naming; StrI, Stroop interference; StrW, Stroop word reading; TB/A, Trail Making Test B/A; VbL, Verbal Learning; VbR, Verbal Recall.

**Fig. S3**


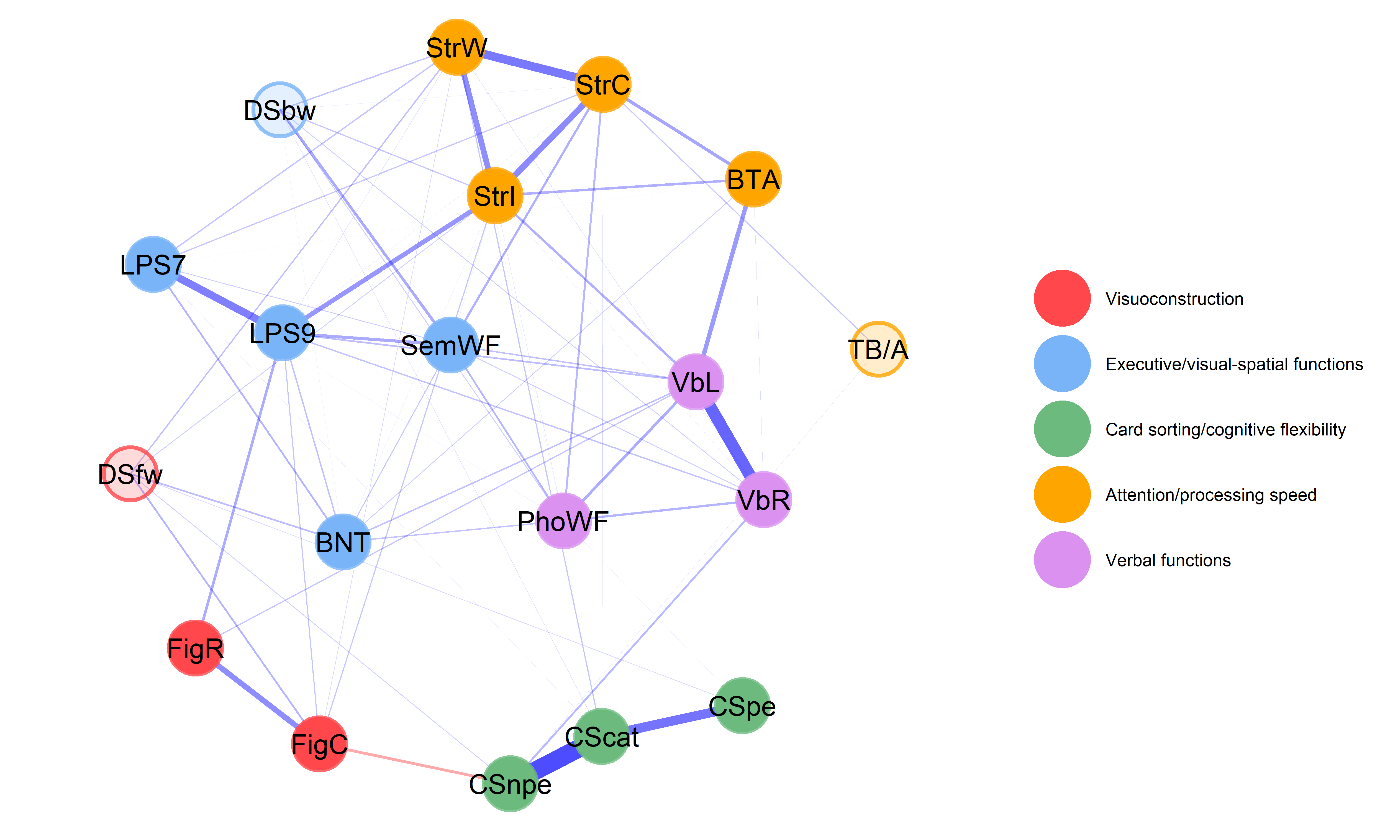


Network and dimensionality structure of residualized cognitive test scores (controlled for study center) in individuals with PD resulting from dynamic exploratory graph analysis. Blue edges indicate positive pairwise conditional associations; red edges indicate negative conditional associations. Node colors indicate assignment to dimensions as empirically derived by dynamic exploratory graph analysis. Transparent node color indicates network loadings below the cut-off for relevancy (.100) in the original dynEGA solution. Dimension names were assigned by the authors based on mutual task features of cognitive test scores within dimensions. Abbreviations: BNT, Boston Naming Test; BTA, Brief Test of Attention; CScat, Modified Card Sorting Test categories; CSnpe, Modified Card Sorting Test non-perservative errors; CSpe, Modified Card Sorting Test perservative errors; DSbw, Digit Span backwards; DSfw, Digit Span forward; EGA, exploratory graph analysis; FigC, Figures Copy; FigR, Figures Recall; LPS7, Leistungsprüfsystem 7; LPS9, Leistungsprüfsystem 9; PhoWF, phonematic Word Fluency; SemWF, semantic Word Fluency; StrC, Stroop color naming; StrI, Stroop interference; StrW, Stroop word reading; TB/A, Trail Making Test B/A; VbL, Verbal Learning; VbR, Verbal Recall.

**Fig. S4**
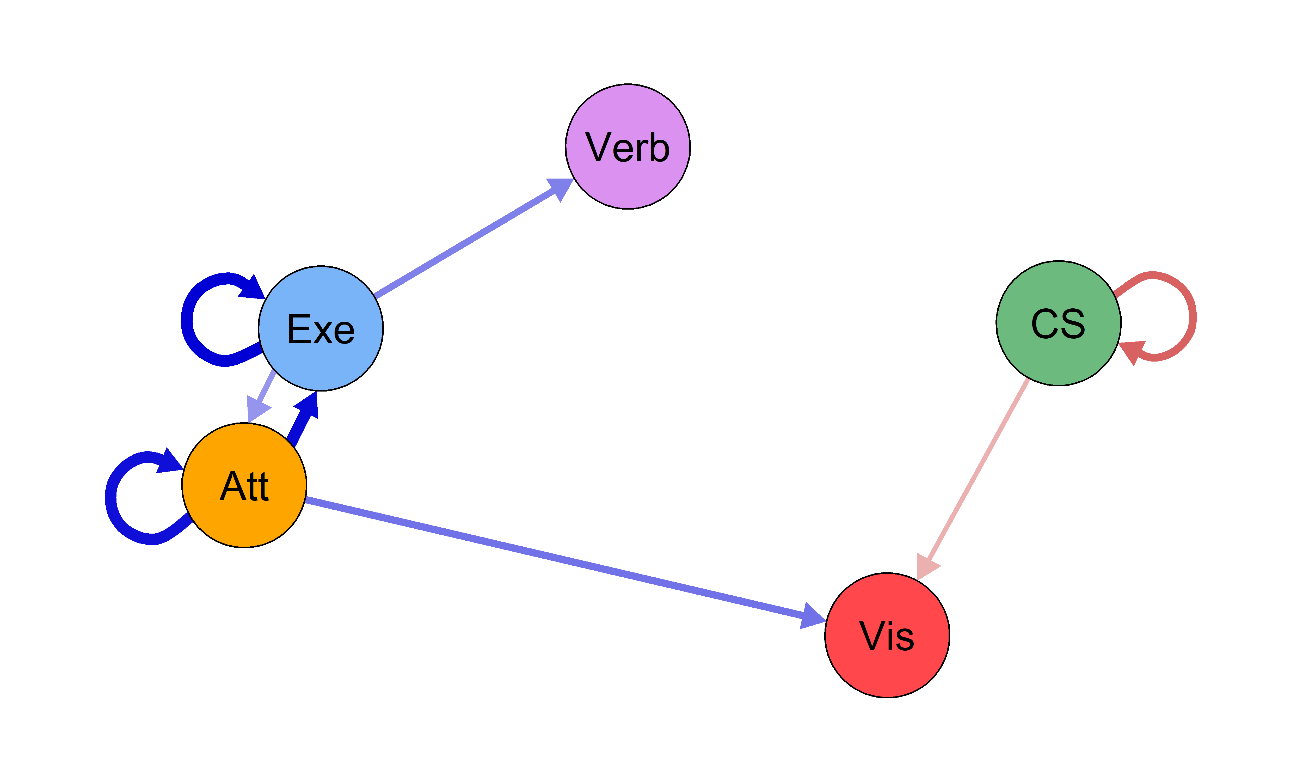


Temporal within-subject effect network model of cognitive dimensions derived by dynamic exploratory graph analysis for a subgroup of people that showed cognitive impairment at baseline assessment (n = 170). Blue edges indicate positive pairwise conditional associations; red edges indicate negative conditional associations. Node colors refer to dimensions identified by dynamic exploratory graph analysis as represented in Figure 1).
Abbreviations: Att, dimension *attention/processing speed*; CS, dimension *card sorting/cognitive flexibility*; Exe, dimension *executive/visual-spatial functions*; Verb, dimension *verbal functions*; Vis, dimension *visuoconstruction*.

**Fig. S5**


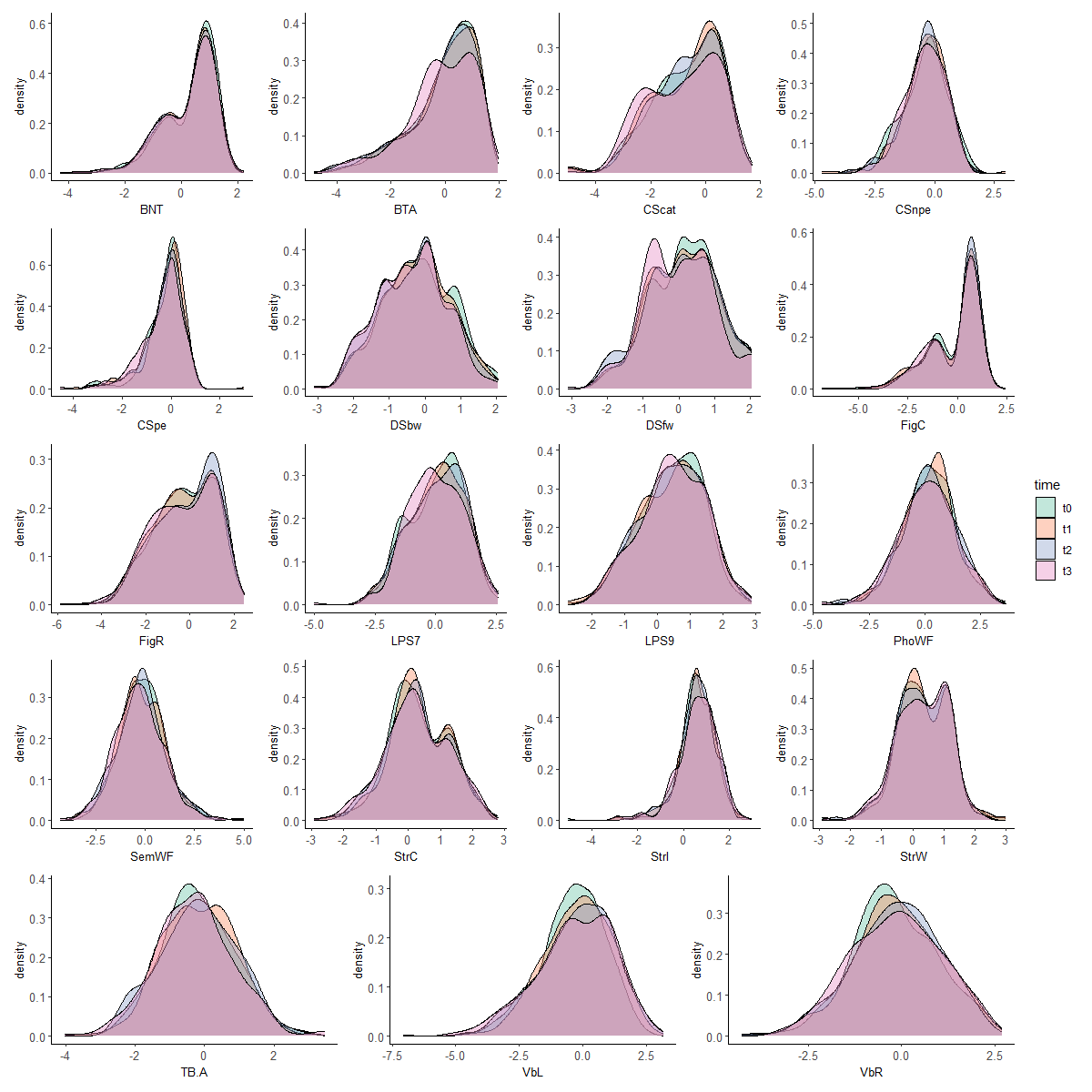


Cognitive test score distributions of different time points.
Abbreviations: BNT, Boston Naming Test; BTA, Brief Test of Attention; CScat, Modified Card Sorting Test categories; CSnpe, Modified Card Sorting Test non-perservative errors; CSpe, Modified Card Sorting Test perservative errors; DSbw, Digit Span backwards; DSfw, Digit Span forward; EGA, exploratory graph analysis; FigC, Figures Copy; FigR, Figures Recall; LPS7, Leistungsprüfsystem 7; LPS9, Leistungsprüfsystem 9; PhoWF, phonematic Word Fluency; SemWF, semantic Word Fluency; StrC, Stroop color naming; StrI, Stroop interference; StrW, Stroop word reading; TB/A, Trail Making Test B/A; VbL, Verbal Learning; VbR, Verbal Recall.

**Fig. S6**


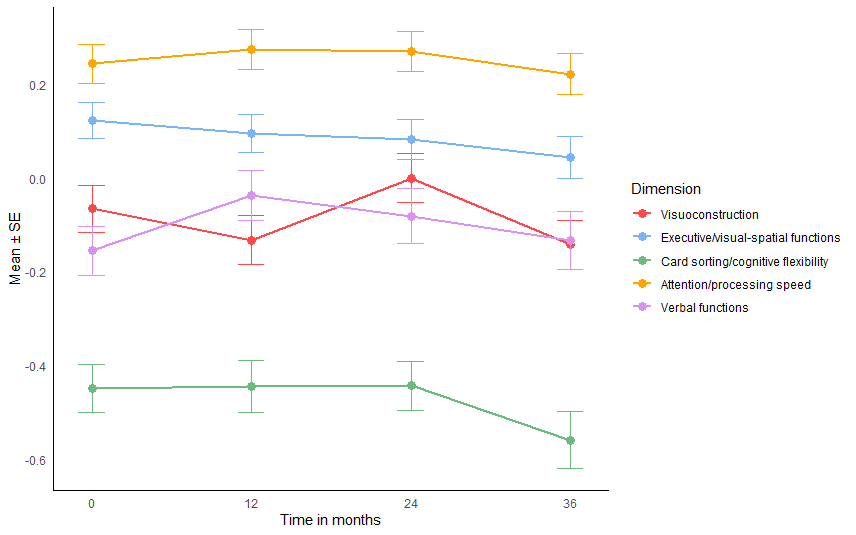


Trajectories of dimension-weighted means of z-standardized cognitive test scores.

**Fig. S7**

**
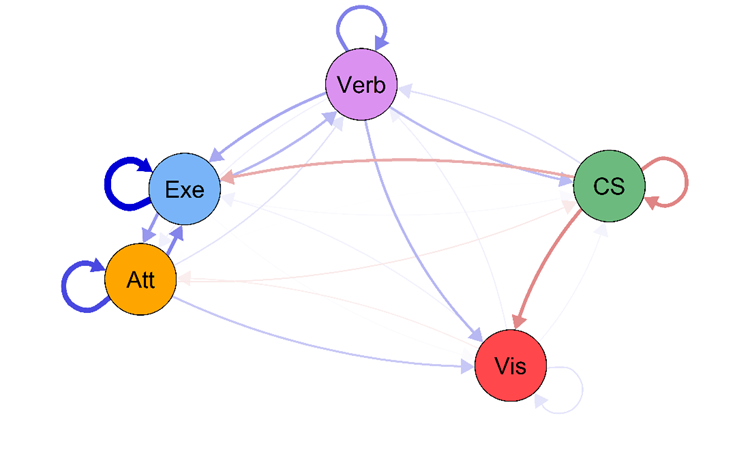
**

Saturated (unpruned) temporal within-subject effect network model of cognitive dimensions derived by dynamic exploratory graph analysis. Blue edges indicate positive pairwise conditional associations; red edges indicate negative conditional associations. Node colors refer to dimensions identified by dynamic exploratory graph analysis as represented in Figure 1.
Abbreviations: Att, dimension *attention/processing speed*; CS, dimension *card sorting/cognitive flexibility*; Exe, dimension *executive/visual-spatial functions*; Verb, dimension *verbal functions*; Vis, dimension *visuoconstruction*.

**Fig. S8**


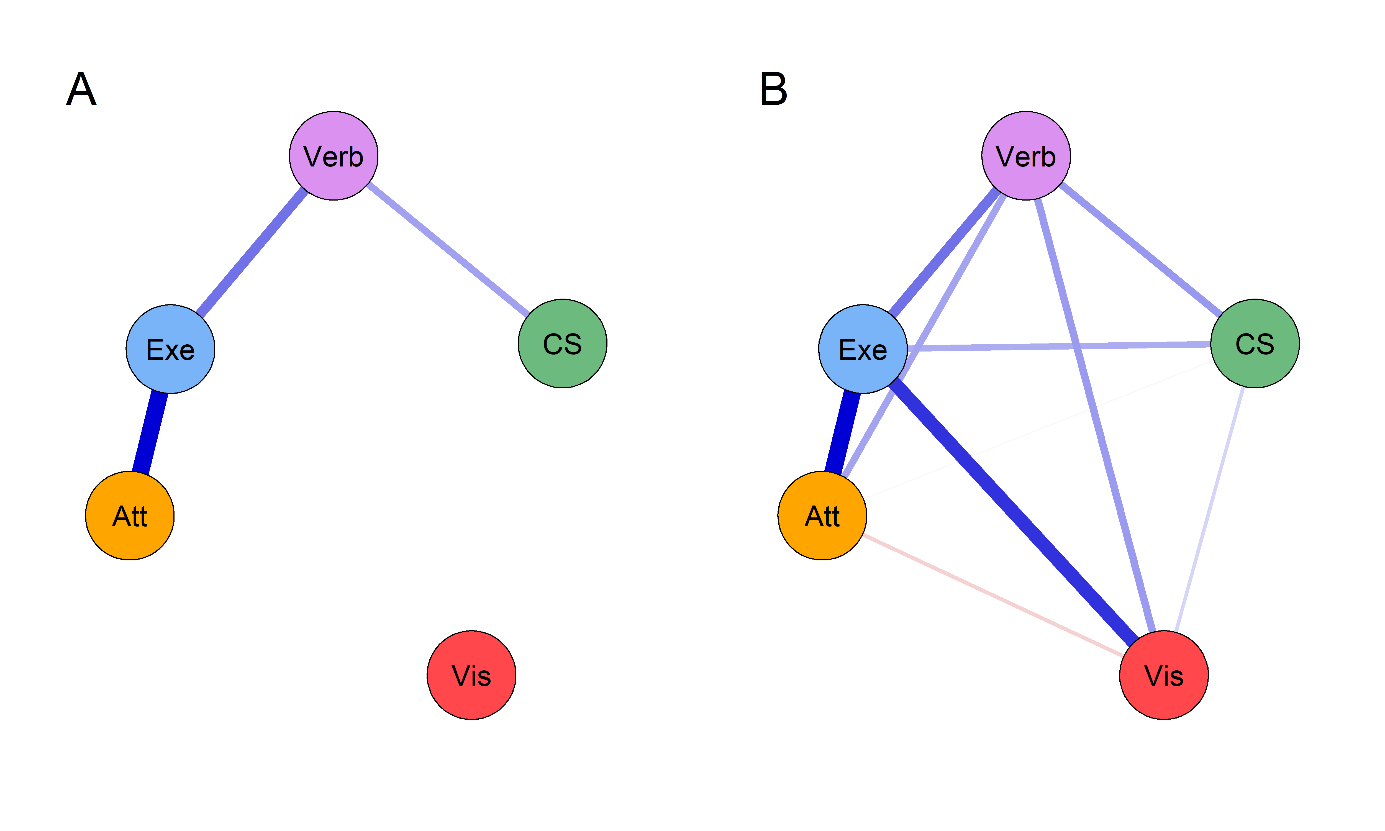


Contemporaneous fixed effect within-subject network model (A) and between-subject network model (B) of cognitive dimensions derived by dynamic exploratory graph analysis. Edges in the contemporaneous model indicates if two variables predict one another within the same measurement occasion after taking temporal information into account. The between-subject network model represents relationships between stable means. Blue edges indicate positive pairwise conditional associations; red edges indicate negative conditional associations. Node colors refer to dimensions identified by dynamic exploratory graph analysis as represented in Figure 1).
Abbreviations: Att, dimension *attention/processing speed*; CS, dimension *card sorting/cognitive flexibility*; Exe, dimension *executive/visual-spatial functions*; Verb, dimension *verbal functions*; Vis, dimension *visuoconstruction*.

**SUPPLEMENTARY TABLES**

**Table S1***. Descriptive characteristics of the sample at baseline separately for cognitive status groups.*

| **Variable** | **PD-NC (n = 185)** | **PD-MCI (n = 145)** | **PD-D (n = 25)** |
| --- | --- | --- | --- |
| Age in years | 65.15 (7.94)  [45-80] | 66.57 (7.59) [45-80] | 72.12 (3.72) [61-78] |
| Gender  *Male*  *Female* | 127 (69.6%)   58 (31.4%) | 102 (70.3%)   43 (29.7%) | 16 (64.0%)   9 (36.0%) |
| Years of education | 14.17 (3.24) [8-20] | 13.47 (2.77) [8-20] | 11.96 (3.36) [8-20] |
| Disease duration in months | 60.55 (46.38) [0-208] | 74.57 (57.64) [0-256] | 125.00 (89.23) [1-297] |
| Levodopa equivalent daily dose | 620.49 (409.34) [52-1837.50] | 743.89 (487.64) [78-2676.50] | 713.20 (416.58) [22.20-1645] |
| UPDRS-III | 19.31 (10.10) [1-50] | 21.15 (10.58) [3-55] | 28.10 (9.32) [14-46] |
| Hoehn & Yahr stages |  |  |  |
| *1* | 38 (20.5%) | 31 (21.4%) | 0 (0.0%) |
| *2* | 117 (63.2%) | 64 (44.1%) | 11 (44.0%) |
| *3* | 25 (13.5%) | 43 (29.7%) | 10 (40.0%) |
| *4* | 4 (2.2%) | 6 (4.1%) | 4 (16.0%) |
| *unknown* | 1 (0.5%) | 1 (0.7%) | 0 (0.0%) |
| Motor phenotype |  |  |  |
| Tremor dominant | 20 (11.0%) | 14 (9.9%) | 1 (4.3%) |
| akinetic rigid | 148 (81.8%) | 112 (79.4%) | 19 (82.6%) |
| Not determined | 13 (7.2%) | 15 (10.6%) | 3 (13.0%) |
| Missing | 4 (2.2%) | 4 (2.8%) | 2 (8.0%) |
| GDS | 2.73 (2.71) [0-14] | 3.08 (2.61) [0-12] | 5.44 (3.24) [0-12] |
| MMSE | 28.96 (1.36) [19-30] | 28.26 (1.58) [22-30] | 24.24 (5.58) [18-29] |
| PANDA | 24.93 (4.02) [13-30] | 22.1 (4.8) [8-30] | 12.72 (5.58) [3-23] |

*Note.* Data are mean (standard deviation) or *n* (%) as appropriate. PD-NC = Parkinson’s Disease normal cognition; PD-MCI = Parkinson’s Disease mild cognitive impairment; PD-D = Parkinson’s Disease dementia; MMSE = Mini-Mental State Examination; PANDA = Parkinson Neuropsychometric Dementia Assessment; UPDRS-III = Unified Parkinson’s Disease Rating Scale Part III; GDS = Geriatric Depression Scale.

**Table S2***. Descriptive characteristics of the sample at baseline separately for motor phenotypes.*

| **Variable** | **TR-D (n = 35)** | **PIGD-D (n = 279)** | **ND (n = 31)** |
| --- | --- | --- | --- |
| Age in years | 64.6 (8.42)  [45-76] | 66.48 (7.63) [45-80] | 65.42 (8.13) [46-80] |
| Gender  *Male*  *Female* | 18 (51.4%)   17 (48.6%) | 198 (71.0%)   81 (29.0%) | 21 (67.7%)   10 (32.3%) |
| Years of education | 13.06 (2.82) [8-19] | 13.74 (3.11) [8-20] | 13.9 (3.61) [8-20] |
| Disease duration in months | 43.29 (44.96) [1-204] | 75.12 (57.55) [0-297] | 58.13 (49.91) [2-190] |
| Levodopa equivalent daily dose | 497.03 (434.09) [52-1964.5] | 704.36 (441.17) [22.2-2676.50] | 575.84 (444.47) [80-2364.6] |
| UPDRS-III | 16.32 (9.55) [4-48] | 21.22 (10.38) [2-55] | 19.97 (11.14) [1-41] |
| Hoehn & Yahr stages |  |  |  |
| *1* | 12 (34.3%) | 46 (16.5%) | 8 (25.8%) |
| *2* | 17 (48.6%) | 157 (56.3%) | 16 (51.6%) |
| *3* | 6 (17.1%) | 62 (22.2%) | 6 (19.4%) |
| *4* | 0 (0.0%) | 12 (4.3%) | 1 (3.2%) |
| *unknown* | 0 (0.0%) | 2 (0.7%) | 0 (0.0%) |
| GDS | 2.14 (2.10) [0-8] | 3.16 (2.73) [0-13] | 3.35 (3.74) [0-14] |
| MMSE | 28.79 (1.59) [23-30] | 28.31 (1.93) [18-30] | 28.55 (2.61) [19-30] |
| PANDA | 22.89 (4.57) [13-30] | 22.68 (5.42) [5-30] | 25.48 (4.44) [13-30] |
| Cognitive status |  |  |  |
| *PD-NC* | 20 (57.1%) | 148 (53.0%) | 12 (41.9%) |
| *PD-MCI* | 14 (40.0%) | 112 (40.1%) | 15 (48.4%) |
| *PDD* | 1 (2.9%) | 19 (6.8%) | 3 (9.7%) |

*Note.* Data are mean (standard deviation) or *n* (%) as appropriate. TR-D = tremor dominant motor phenotype; PIGD-D = akinetic-rigid motor phenotype with postural instability and gait difficulty; ND = not determined motor phenotype with mixed symptoms; MMSE = Mini-Mental State Examination; PANDA = Parkinson Neuropsychometric Dementia Assessment; UPDRS-III = Unified Parkinson’s Disease Rating Scale Part III; GDS = Geriatric Depression Scale. PD-NC = Parkinson’s Disease normal cognition; PD-MCI = Parkinson’s Disease mild cognitive impairment; PD-D = Parkinson’s Disease dementia.

**Table S3*.*** *Network loadings for cognitive dimensions identified by dynamic exploratory graph analysis.*

|  | Dimension | | | | |
| --- | --- | --- | --- | --- | --- |
| Test variable | Visuo-construction | Executive /visual-spatial functions | Card sorting/ cognitive flexibility | Attention/ processing speed | Verbal functions |

| FigC | **.248** |  |  |  |  |
| --- | --- | --- | --- | --- | --- |
| FigR | **.180** |  |  |  |  |
| DSfw | **.067*** |  |  |  |  |
| LPS7 |  | **.224** |  |  |  |
| LPS9 | *.129* | **.253** |  | *.105* |  |
| SemWF |  | **.171** |  |  | *.106* |
| BNT |  | **.103** |  |  |  |
| DSbw |  | **.063*** |  |  |  |
| CScat |  |  | **.602** |  |  |
| CSnpe | *.122* |  | **.396** |  |  |
| CSpe |  |  | **.206** |  |  |
| StrW |  |  |  | **.272** |  |
| StrC |  |  |  | **.362** |  |
| StrI |  | *.131* |  | **.273** |  |
| BTA |  |  |  | **.110** | *.109* |
| TB/A |  |  |  | **.022*** |  |
| VbL |  |  |  | *.130* | **.335** |
| VbR |  |  |  |  | **.326** |
| PhoWF |  |  |  |  | **.114** |

*Note*. Relevant network loadings (< .100) for the cognitive dimensions identified by dynamic exploratory graph analysis. Values marked in bold indicate that the test score was assigned to the corresponding dimension by dynamic exploratory graph analysis. Values marked in italics indicate potential cross-loadings with other dimensions. * Assigned to domain, although network loading < .100.
Abbreviations: DSfw = Digit Span forward; DSbw = Digit Span backwards; SemWF = semantic word fluency; PhoWF = phonematic word fluency; TB/A = Trail Making Test B/A; FigC = Figures Copy; FigR = Figures Recall; LPS7 = Leistungsprüfsystem 7; LPS9 = Leistungsprüfsystem 9; BNT = Boston Naming Test; VbL = verbal learning; VbR = verbal recall; CScat = Modified Card Sorting Test categories; CSnpe = Modified Card Sorting Test non-perservative errors; CSpe = Modified Card Sorting Test perservative errors; BTA = Brief Test of Attention; StrW = Stroop word reading; StrC = Stroop color naming; StrI = Stroop interference.

**Table S4***. Task features of cognitive subtasks included in the analyses.*

| Type/Feature | CERAD Figures Copy  (FigC) | CERAD Figures Recall  (FigR) | Digit Span Forward (DSfw) | LPS50+ Subtest 7 Spatial Rotation  (LPS7) | LPS50+ Subtest 9 Spatial Reasoning (LPS9) | Semantic Verbal Fluency  (SemWF) | CERAD Boston Naming Test  (BNT) | Digit Span Backward  (DSbw) | MCST Category  (CScat) | MCST non-persevera-tive errors  (CSnpe) | MCST persevera-tive errors  (CSpe) | Stroop Word Reading  (StrW) | Stroop Color Naming  (StrC) | Stroop Interference  (StrI) | Brief Test of Attention  (BTA) | CERAD Trail Making Test B/A  (TB/A) | CERAD Verbal Learning  (VbL) | CERAD Verbal Recall  (VbR) | CERAD Phonematic Word Fluency  (PhoWF) |
| --- | --- | --- | --- | --- | --- | --- | --- | --- | --- | --- | --- | --- | --- | --- | --- | --- | --- | --- | --- |
| **PROCESS** |  |  |  |  |  |  |  |  |  |  |  |  |  |  |  |  |  |  |  |
| Memory (Free Recall) | 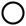 | 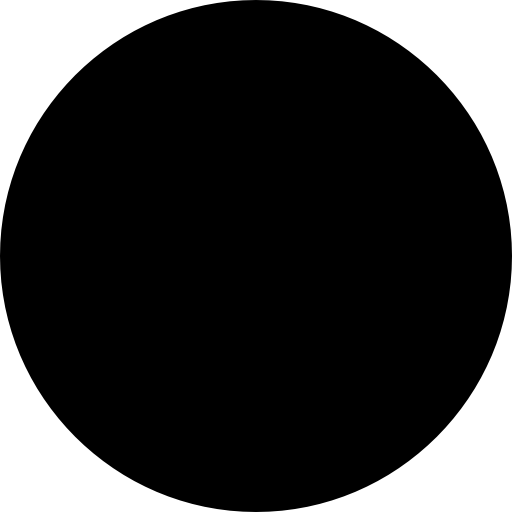 | 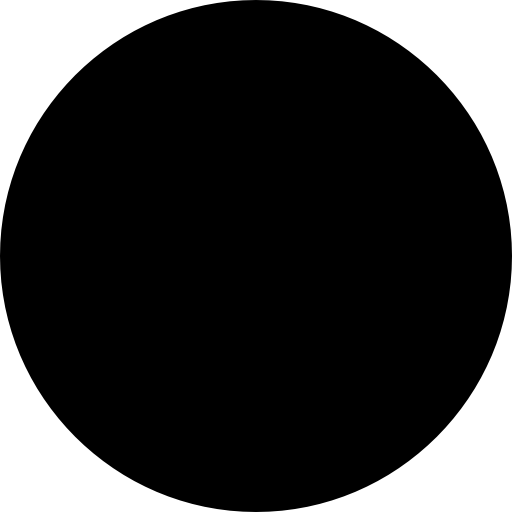 | 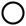 | 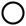 | 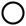 | 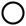 | 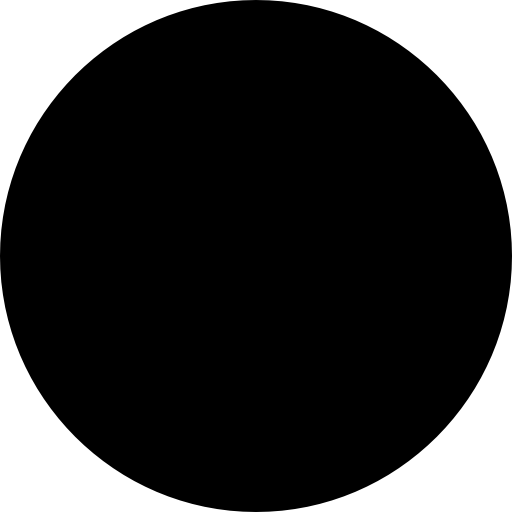 | 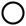 | 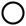 | 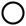 | 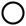 | 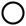 | 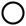 | 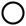 | 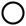 | 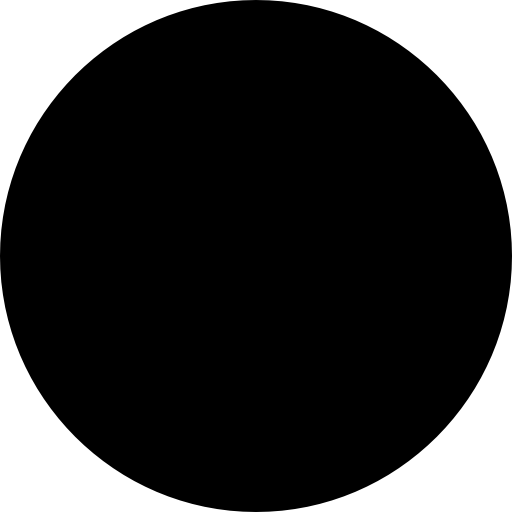 | 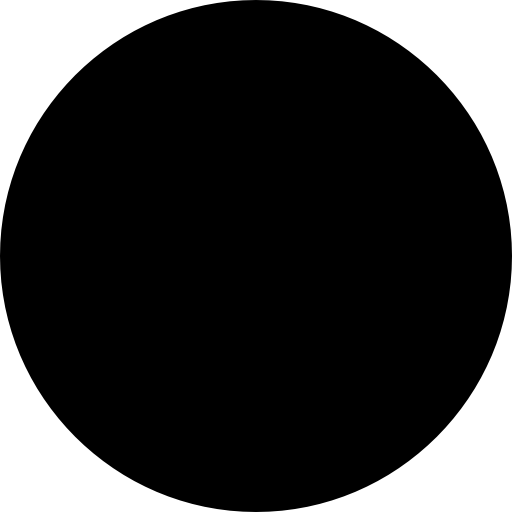 | 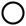 |
| Semantic Memory | 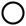 | 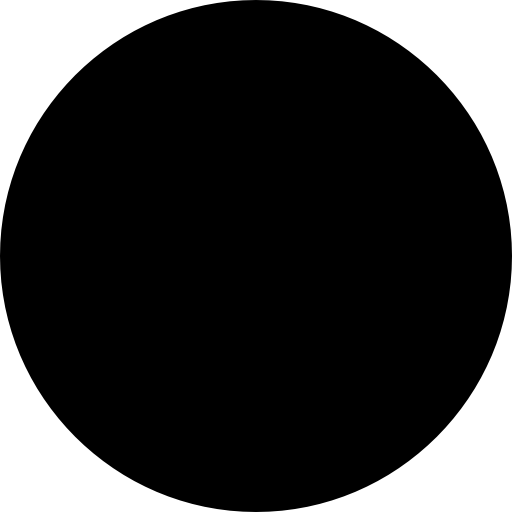 | 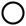 | 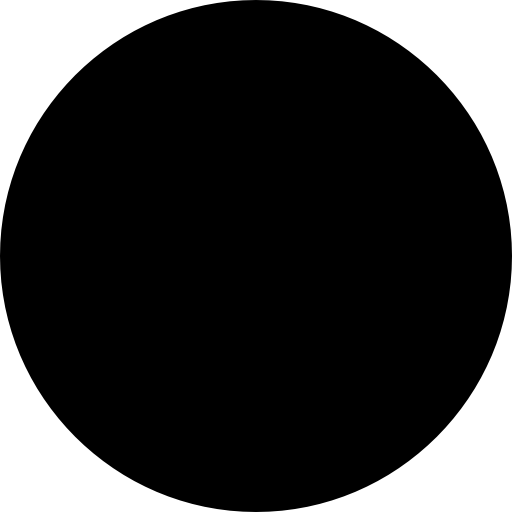 | 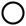 | 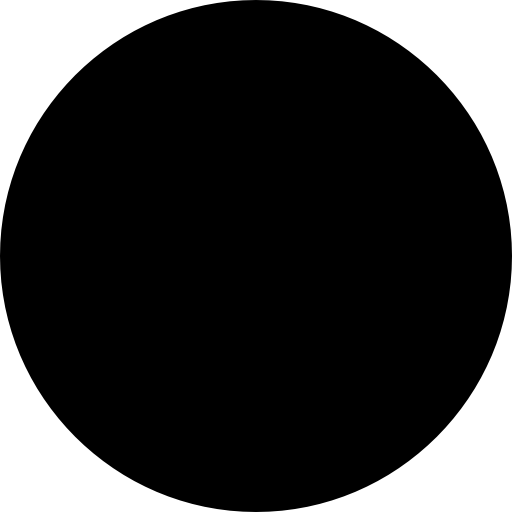 | 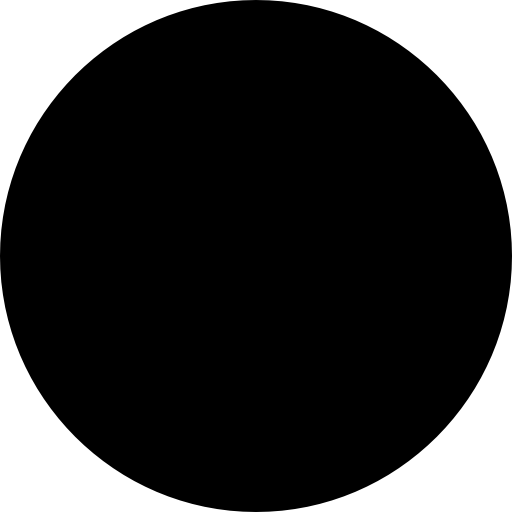 | 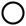 | 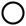 | 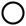 | 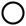 | 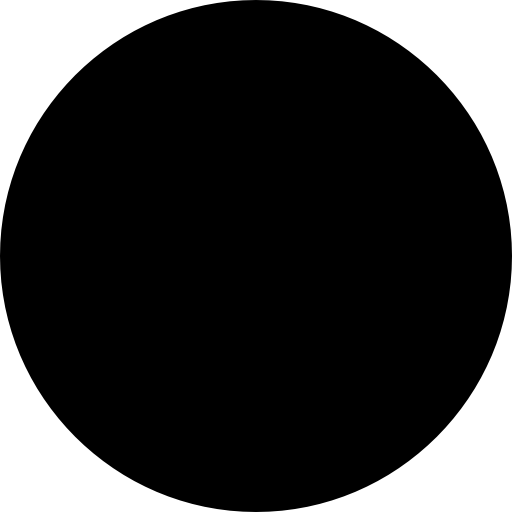 | 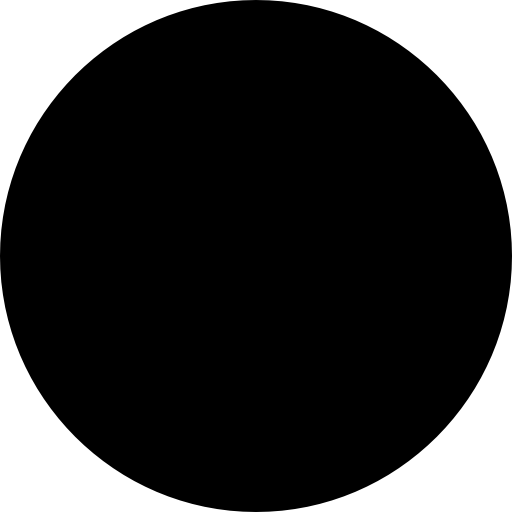 | 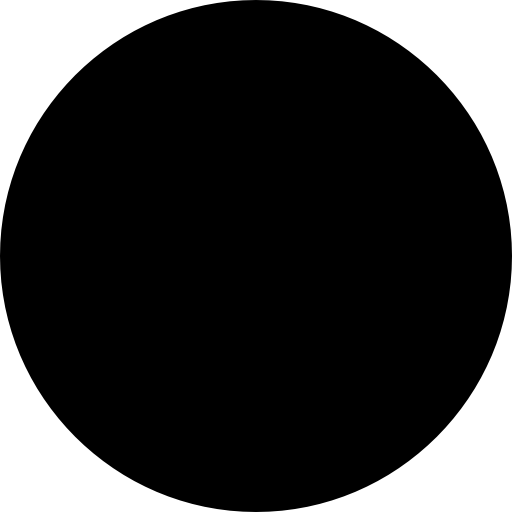 | 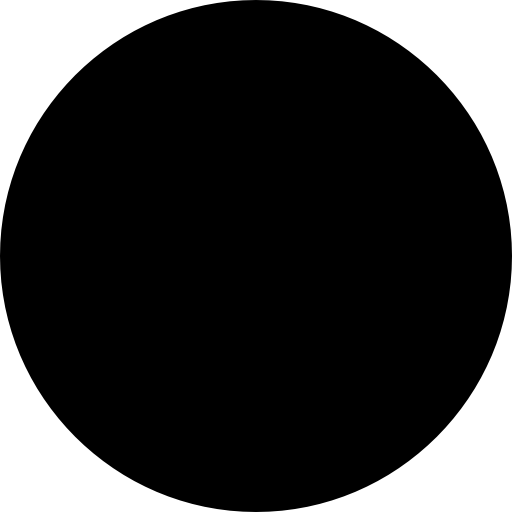 | 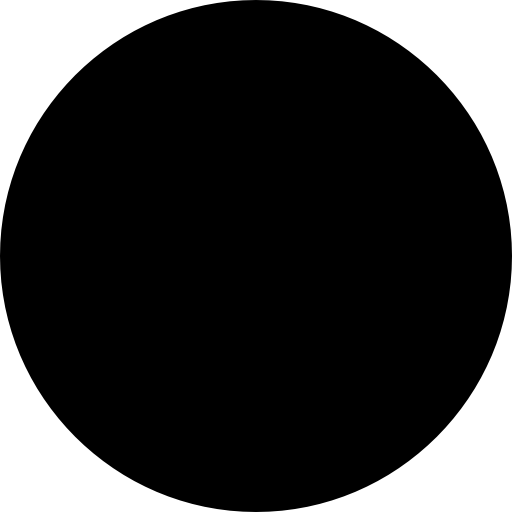 | 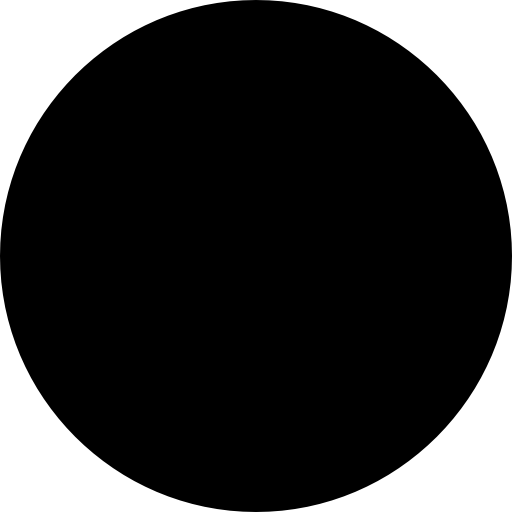 | 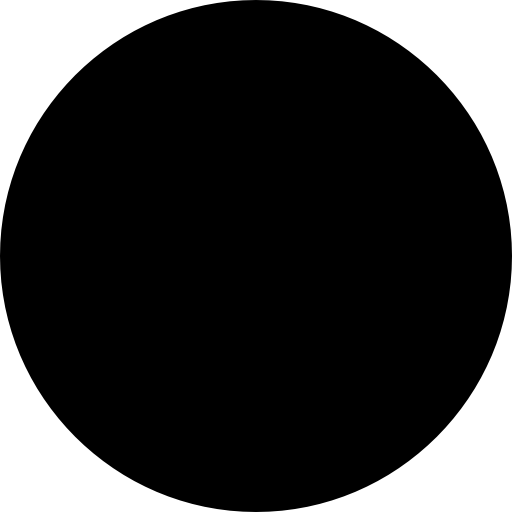 | 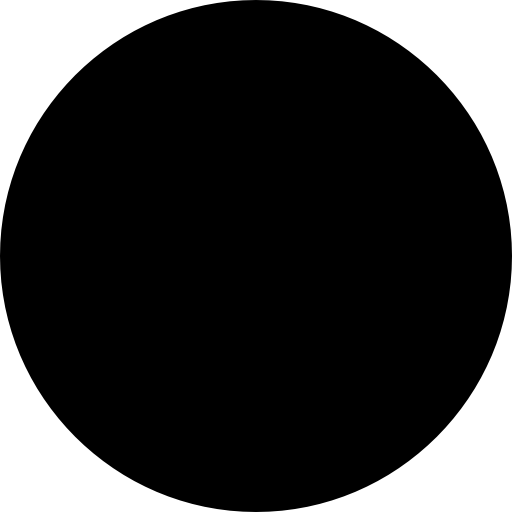 |
| Perceptual Memory | 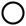 | 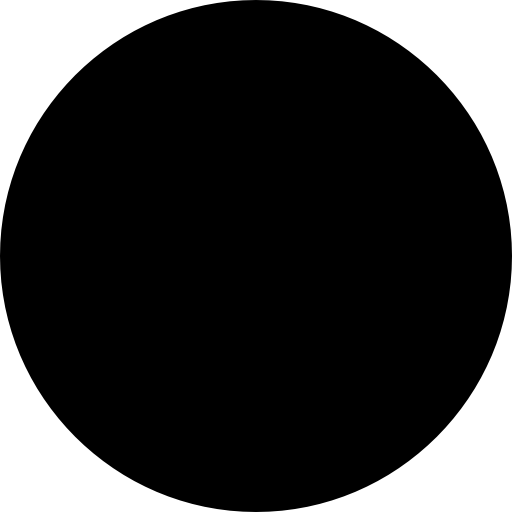 | 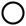 | 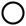 | 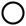 | 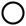 | 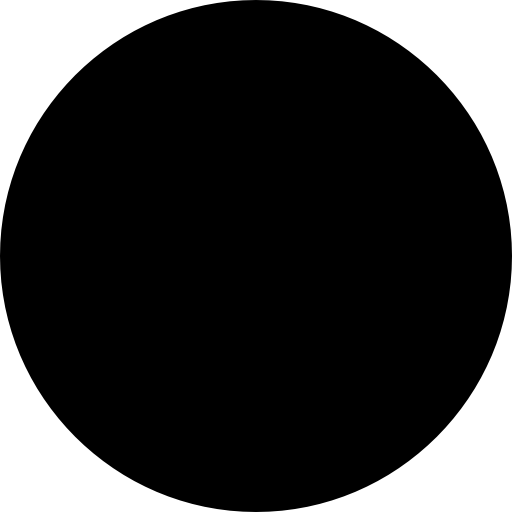 | 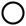 | 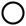 | 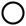 | 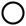 | 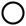 | 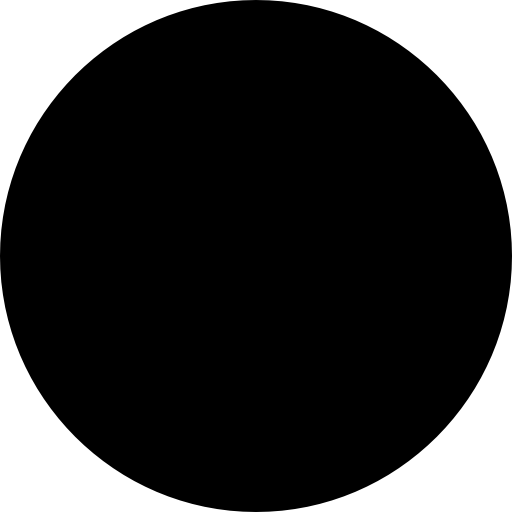 | 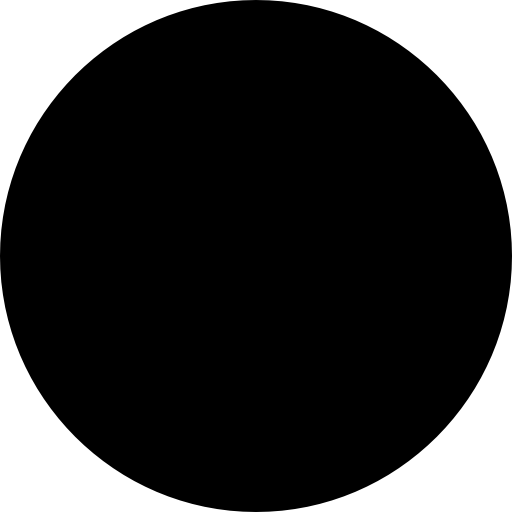 | 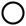 | 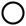 | 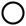 | 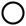 | 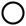 |
| Memory Updating | 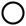 | 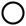 | 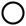 | 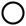 | 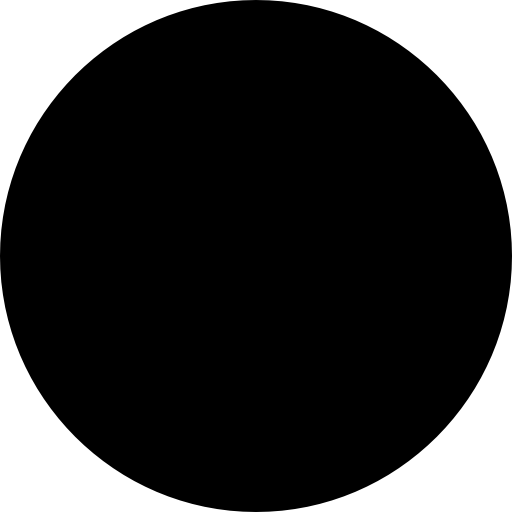 | 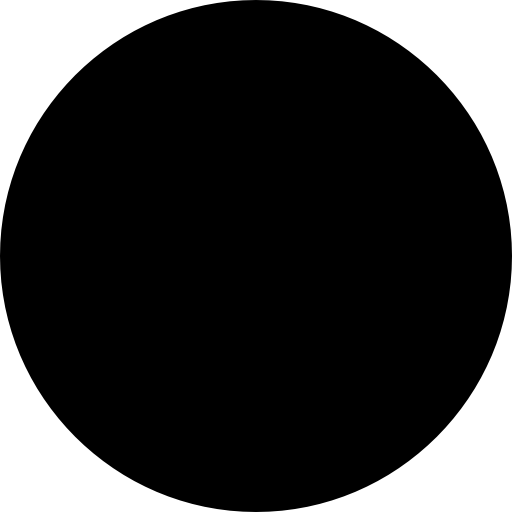 | 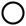 | 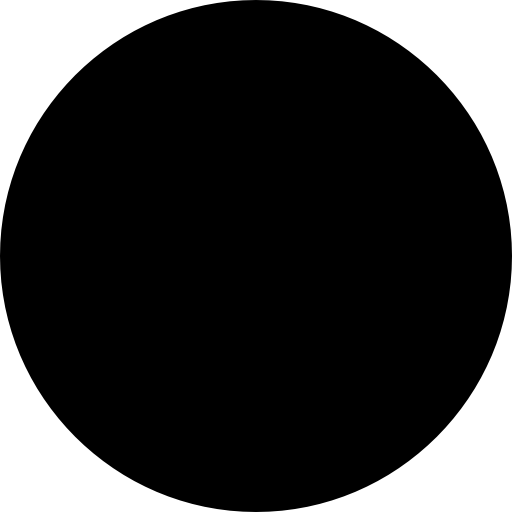 | 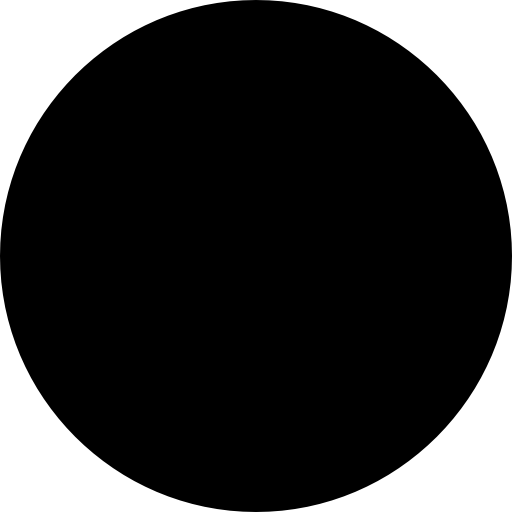 | 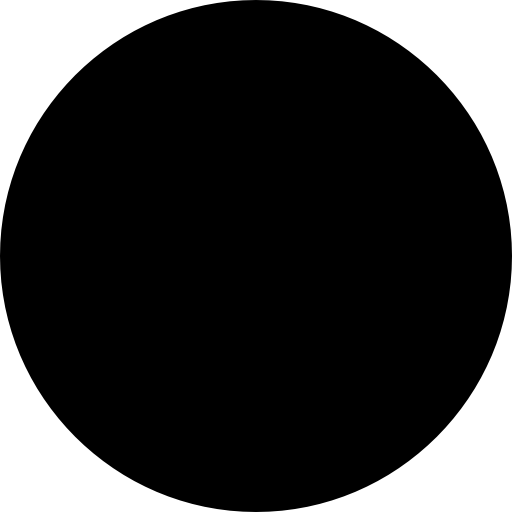 | 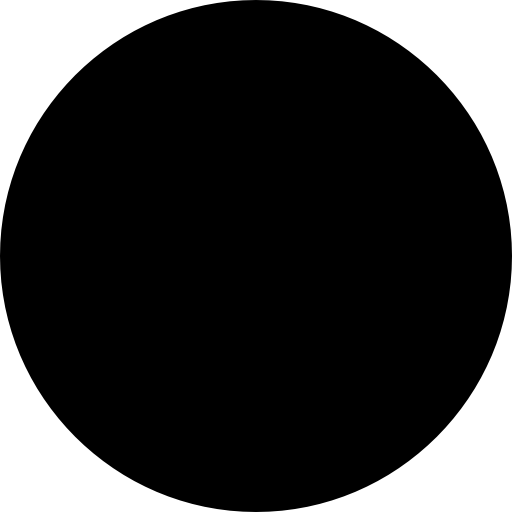 | 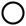 | 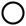 | 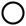 | 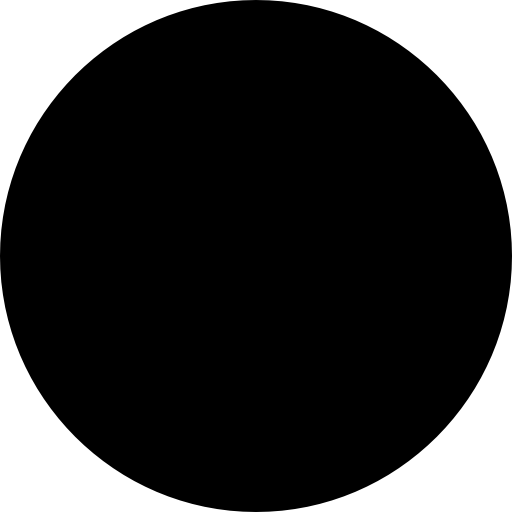 | 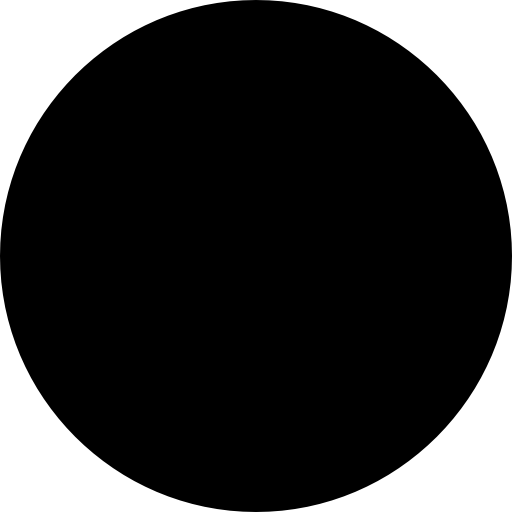 | 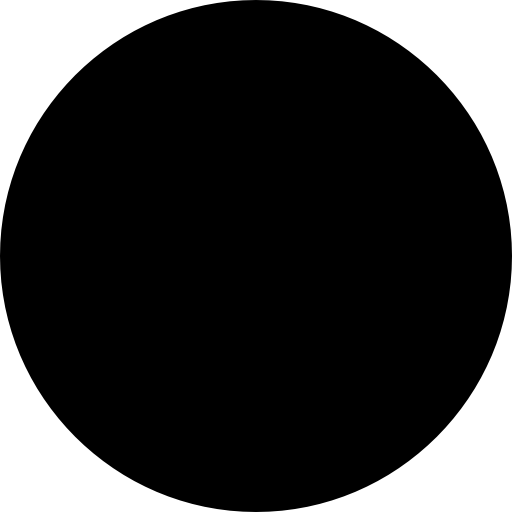 | 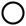 | 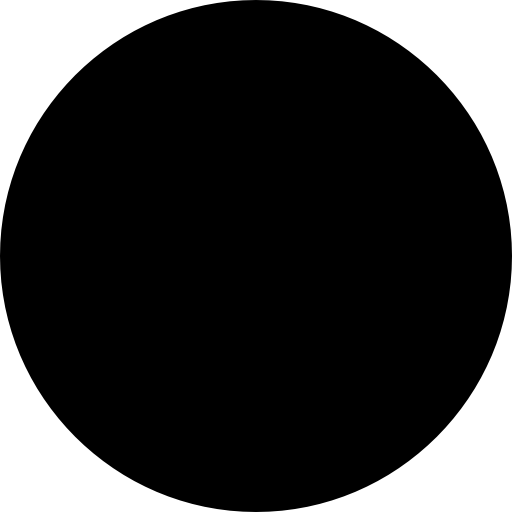 |
| Selective Attention | 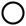 | 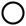 | 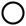 | 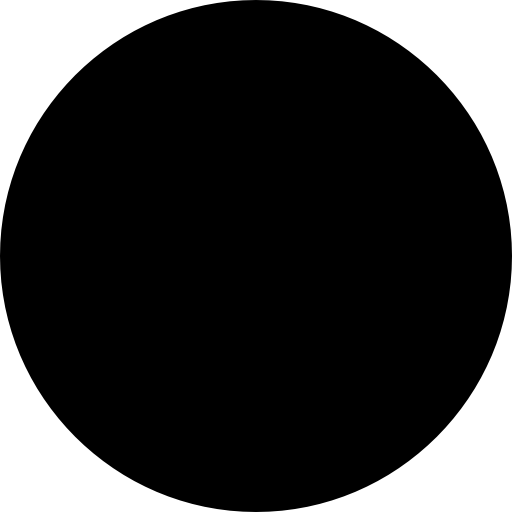 | 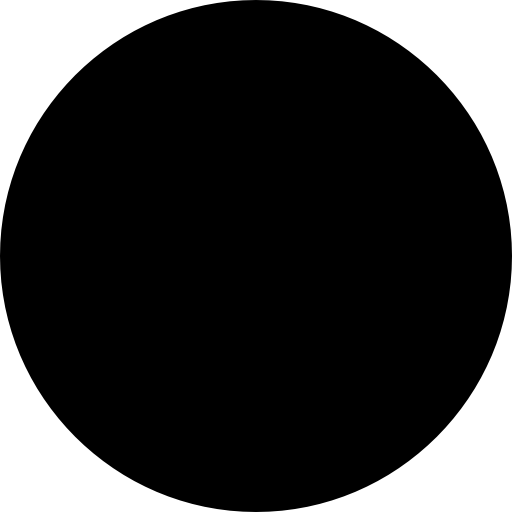 | 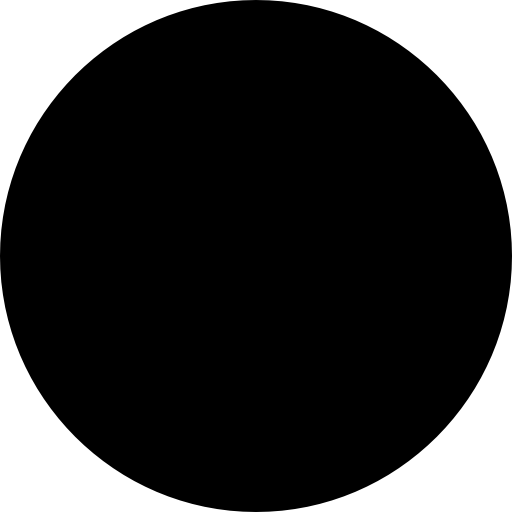 | 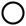 | 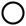 | 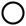 | 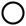 | 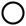 | 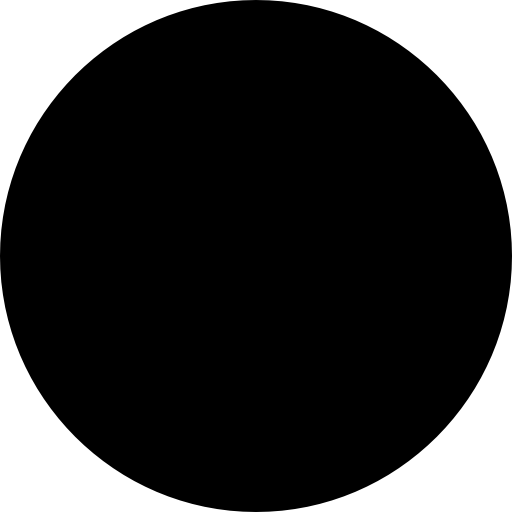 | 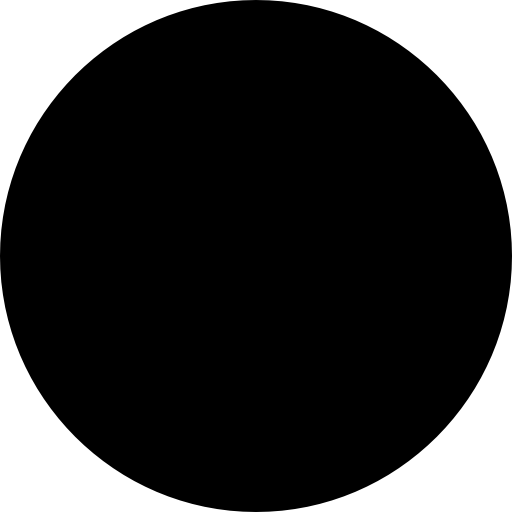 | 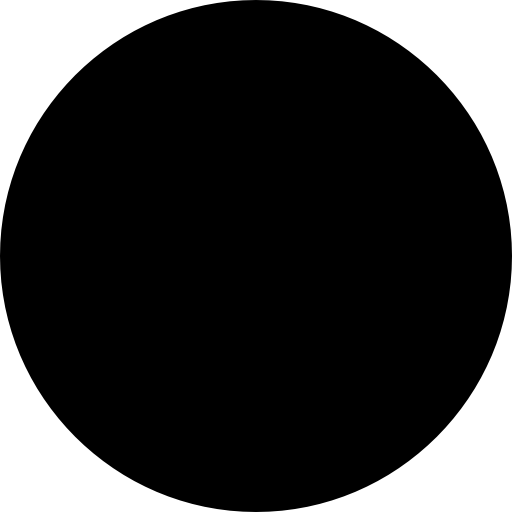 | 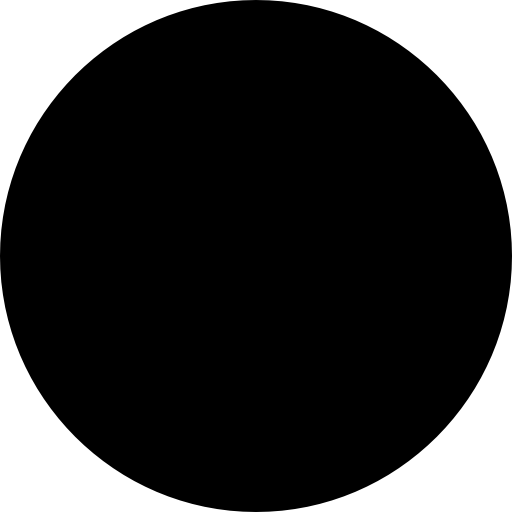 | 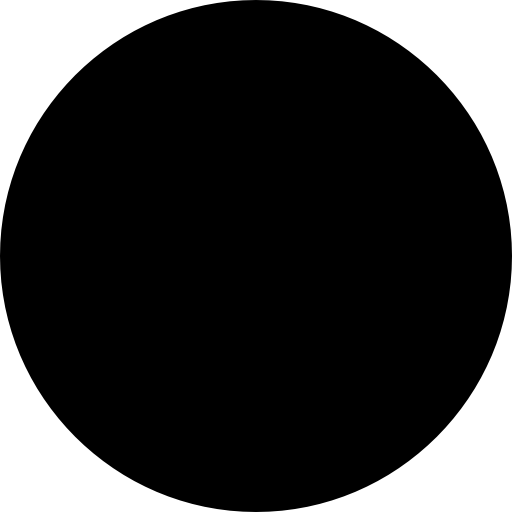 |  |  |  |
| Divided Attention |  |  |  |  |  |  |  |  |  |  |  |  |  |  |  |  |  |  |  |
| Sustained Attention |  |  |  |  |  |  |  |  |  |  |  |  |  |  |  |  |  |  |  |
| Set Shifting |  |  |  |  |  |  |  |  |  |  |  |  |  |  |  |  |  |  |  |
| Vocabulary Knowledge |  |  |  |  |  |  |  |  |  |  |  |  |  |  |  |  |  |  |  |
| Word Generation |  |  |  |  |  |  |  |  |  |  |  |  |  |  |  |  |  |  |  |
| Planning |  |  |  |  |  |  |  |  |  |  |  |  |  |  |  |  |  |  |  |
| Spatial Reasoning |  |  |  |  |  |  |  |  |  |  |  |  |  |  |  |  |  |  |  |
| Logical Reasoning |  |  |  |  |  |  |  |  |  |  |  |  |  |  |  |  |  |  |  |
| Response Inhibition |  |  |  |  |  |  |  |  |  |  |  |  |  |  |  |  |  |  |  |
| Motor Praxis |  |  |  |  |  |  |  |  |  |  |  |  |  |  |  |  |  |  |  |
| **STIMULUS** |  |  |  |  |  |  |  |  |  |  |  |  |  |  |  |  |  |  |  |
| Objects |  |  |  |  |  |  |  |  |  |  |  |  |  |  |  |  |  |  |  |
| Locations |  |  |  |  |  |  |  |  |  |  |  |  |  |  |  |  |  |  |  |
| Single Letters |  |  |  |  |  |  |  |  |  |  |  |  |  |  |  |  |  |  |  |
| Words |  |  |  |  |  |  |  |  |  |  |  |  |  |  |  |  |  |  |  |
| Digits |  |  |  |  |  |  |  |  |  |  |  |  |  |  |  |  |  |  |  |
| Numbers |  |  |  |  |  |  |  |  |  |  |  |  |  |  |  |  |  |  |  |
| **STIMULUS MODALITY** |  |  |  |  |  |  |  |  |  |  |  |  |  |  |  |  |  |  |  |
| Visual |  |  |  |  |  |  |  |  |  |  |  |  |  |  |  |  |  |  |  |
| Auditory |  |  |  |  |  |  |  |  |  |  |  |  |  |  |  |  |  |  |  |
| **RESPONSE** |  |  |  |  |  |  |  |  |  |  |  |  |  |  |  |  |  |  |  |
| Objects |  |  |  |  |  |  |  |  |  |  |  |  |  |  |  |  |  |  |  |
| Locations |  |  |  |  |  |  |  |  |  |  |  |  |  |  |  |  |  |  |  |
| Single Letters |  |  |  |  |  |  |  |  |  |  |  |  |  |  |  |  |  |  |  |
| Words |  |  |  |  |  |  |  |  |  |  |  |  |  |  |  |  |  |  |  |
| Digits |  |  |  |  |  |  |  |  |  |  |  |  |  |  |  |  |  |  |  |
| Numbers |  |  |  |  |  |  |  |  |  |  |  |  |  |  |  |  |  |  |  |
| **RESPONSE MODALITY** |  |  |  |  |  |  |  |  |  |  |  |  |  |  |  |  |  |  |  |
| Timed |  |  |  |  |  |  |  |  |  |  |  |  |  |  |  |  |  |  |  |
| Speech |  |  |  |  |  |  |  |  |  |  |  |  |  |  |  |  |  |  |  |
| Pencil |  |  |  |  |  |  |  |  |  |  |  |  |  |  |  |  |  |  |  |
| Key press |  |  |  |  |  |  |  |  |  |  |  |  |  |  |  |  |  |  |  |

*Note*. Task features are adapted, modified and amended based on the task feature glossary by Steyvers & Schafer (2020). For details, see Table S4. Solid black circles indicate the presence of the task feature according to expert consensus, hollow circles indicate the absence of the task feature. Solid grey circles indicate the presence of the task feature may depend on the strategy used by an individual to solve the task. Crossed circles indicate that no stimulus is presented during task instruction of the specific subtask, but task solving relies on stimuli introduced during a previous subtask (e.g., free recall of previously learned words).

**Table S5**. *Descriptions of task features.*

| Task Feature | Description |
| --- | --- |
| **PROCESS** |  |
| Memory (Free Recall) | The task requires recall of information that is no longer shown |
| Semantic Memory | The task requires recall of general knowledge about the world, such as facts, concepts, and meaning of words or objects |
| Perceptual Memory | The task requires recalling or recognizing and previously encountered sensory information, such as colors, shapes or sounds |
| Memory Updating | The task involves updating a representation of information as additional stimuli are presented |
| Selective Attention | The task requires attending to some sources of information while ignoring others at the same time |
| Divided Attention | The task requires integration of information from multiple sources presented concurrently |
| Sustained Attention | The task requires maintaining attention on a specific task or stimulus over an extended period of time |
| Set Shifting | The task requires switching between different rules for mapping from the stimulus to the response |
| Vocabulary Knowledge | The task requires knowledge of the meaning of words or semantic relationships between words |
| Word Generation | The task requires producing or identifying words |
| Planning | The task requires selecting and sequencing a set of actions to reach a goal |
| Spatial Reasoning | The task requires perception of relationships between objects in space and reasoning about spatial transformation |
| Logical Reasoning | The task requires recognizing patterns in symbolic information to arrive at unstated conclusions |
| Response Inhibition | The task requires suppression of previously learned mappings between stimulus and response |
| Motor Praxis | The task requires planning and executing motor actions |
| **STIMULUS** |  |
| Objects | Stimulus involves depictions of objects (other than words or numbers) |
| Locations | Spatial positions are the targets of cognitive processes |
| Single Letters | Stimulus involves letters of the alphabet as symbols or for property evaluation (e.g. vowel/consonant) |
| Words | Stimulus involves words |
| Digits | Stimulus involves single digits (0-9) as symbols with no numerical value or for property evaluation (e.g. odd/even) |
| Numbers | Stimulus involves numerical values |
| **STIMULUS MODALITY** |  |
| Visual | Stimulus is presented visually |
| Auditory | Stimulus is presented auditory |
| **RESPONSE** |  |
| Objects | Response involves depictions of objects (other than words or numbers) |
| Locations | Response involves spatial positions |
| Single Letters | Response involves letters of the alphabet as symbols |
| Words | Response involves words |
| Digits | Response involves single digits (0-9) as symbols with no numerical value |
| Numbers | Response involves numerical values |
| **RESPONSE MODALITY** |  |
| Timed | Response has a time constraint or is timed |
| Speech | Response is delivered by speaking out loud |
| Pencil | Response is delivered by using a pen to write, draw or cross out |
| Key Press | Response is delivered by pressing a key on a keyboard |

*Note.* Descriptions of task features are adapted, modified and amended based on the task feature glossary by Steyvers & Schafer (2020).

**Table S6***. Identified number of dimensions in dynamic exploratory graph during subsampling procedures (N = 1000).*

| **Number of identified dimensions** | 3 | 4 | 5 | 6 |
| --- | --- | --- | --- | --- |
| **Proportion** | 0.004 | 0.426 | 0.567 | 0.003 |

**Table S7***. Model comparison for linear mixed-effects model with Mini Mental Status Examination total score as dependent variable.*

| **Model** | ***n*** | ***k*** | **LL** | **BIC** | **AIC** | **ΔAIC** |
| --- | --- | --- | --- | --- | --- | --- |
| dynEGA | 1028 | 11 | -1798.77 | 3673.82 | 3619.54 |  |
| theoretical domains | 1028 | 11 | -1799.94 | 3676.16 | 3621.97 | 2.33 |

*Note.* n = number of observations. k = number of random-effects terms. LL = log-likelihood. BIC = Bayesian information criterion. AIC = Akaike information criterion.

**Table S8.** Model summary for the *linear mixed-effects model with Mini Mental Status Examination total score as dependent variable and time and dynEGA dimensions as predictors.*

|  | **MMSE** | | |
| --- | --- | --- | --- |
| **Predictors** | **Estimates** | **CI** | ***p*** |
| (Intercept) | 28.58 | 28.40 – 28.76 | **<0.001** |
| time [t1] | -0.02 | -0.22 – 0.19 | 0.870 |
| time [t2] | -0.07 | -0.28 – 0.15 | 0.552 |
| time [t3] | -0.01 | -0.23 – 0.21 | 0.919 |
| Visuoconstruction | 0.27 | 0.14 – 0.39 | **<0.001** |
| Executive/visual-spatial functions | 0.28 | 0.08 – 0.48 | **0.006** |
| Card sorting/cognitive flexibility | 0.25 | 0.13 – 0.37 | **<0.001** |
| Attention/processing speed | 0.23 | 0.04 – 0.42 | **0.016** |
| Verbal functions | 0.16 | 0.03 – 0.29 | **0.014** |
| **Random Effects** | | | |
| σ^2^ | 1.54 | | |
| τ_00_ _ID_ | 0.54 | | |
| ICC | 0.26 | | |
| N_ID_ | 320 | | |
| Observations | 1028 | | |
| Marginal R^2^ / Conditional R^2^ | 0.172 / 0.388 | | |

**Table S9.** Model summary for the linear mixed-effects model with Mini Mental Status Examination total score as dependent variable and time and theoretically assumed cognitive domains as predictors*.*

|  | **MMSE** | | |
| --- | --- | --- | --- |
| **Predictors** | **Estimates** | **CI** | ***p*** |
| (Intercept) | 28.40 | 28.21 – 28.59 | **<0.001** |
| time [t1] | -0.01 | -0.22 – 0.19 | 0.910 |
| time [t2] | -0.04 | -0.25 – 0.18 | 0.741 |
| time [t3] | 0.00 | -0.22 – 0.23 | 0.975 |
| Memory | 0.14 | 0.00 – 0.27 | **0.045** |
| Executive functions | 0.33 | 0.13 – 0.52 | **0.001** |
| Attention | 0.37 | 0.19 – 0.56 | **<0.001** |
| Visuospatial functions | 0.18 | 0.04 – 0.32 | **0.010** |
| Language | 0.21 | 0.10 – 0.32 | **<0.001** |
| **Random Effects** | | | |
| σ^2^ | 1.51 | | |
| τ_00_ _ID_ | 0.61 | | |
| ICC | 0.29 | | |
| N_ID_ | 320 | | |
| Observations | 1028 | | |
| Marginal R^2^ / Conditional R^2^ | 0.167 / 0.405 | | |

**Table S10***. Valid and missing pairwise-observations at baseline assessment t0.*

| **Variable** | **DSfw** | **DSbw** | **CScat** | **CSnpe** | **CSpe** | **BTA** | **StrW** | **StrC** | **StrI** | **LPS7** | **LPS9** | **SemWF** | **PhoWF** | **VbL** | **VbR** | **TB/A** | **FigC** | **FigR** | **BNT** |
| --- | --- | --- | --- | --- | --- | --- | --- | --- | --- | --- | --- | --- | --- | --- | --- | --- | --- | --- | --- |
| **DSfw** | 352 (3) | 352 (3) | 299 (56) | 298 (57) | 298 (57) | 332 (23) | 336 (19) | 336 (19) | 335 (20) | 343 (12) | 343 (12) | 351 (4) | 350 (5) | 351 (4) | 351 (4) | 336 (19) | 351 (4) | 350 (5) | 351 (4) |
| **DSbw** | 352 (3) | 352 (3) | 299 (56) | 298 (57) | 298 (57) | 332 (23) | 336 (19) | 336 (19) | 335 (20) | 343 (12) | 343 (12) | 351 (4) | 350 (5) | 351 (4) | 351 (4) | 336 (19) | 351 (4) | 350 (5) | 351 (4) |
| **CScat** | 299 (56) | 299 (56) | 299 (56) | 298 (57) | 298 (57) | 293 (62) | 292 (63) | 292 (63) | 292 (63) | 297 (58) | 297 (58) | 299 (56) | 298 (57) | 299 (56) | 299 (56) | 293 (62) | 299 (56) | 298 (57) | 299 (56) |
| **CSnpe** | 298 (57) | 298 (57) | 298 (57) | 298 (57) | 298 (57) | 292 (63) | 291 (64) | 291 (64) | 291 (64) | 296 (59) | 296 (59) | 298 (57) | 297 (58) | 298 (57) | 298 (57) | 292 (63) | 298 (57) | 297 (58) | 298 (57) |
| **CSpe** | 298 (57) | 298 (57) | 298 (57) | 298 (57) | 298 (57) | 292 (63) | 291 (64) | 291 (64) | 291 (64) | 296 (59) | 296 (59) | 298 (57) | 297 (58) | 298 (57) | 298 (57) | 292 (63) | 298 (57) | 297 (58) | 298 (57) |
| **BTA** | 332 (23) | 332 (23) | 293 (62) | 292 (63) | 292 (63) | 333 (22) | 322 (33) | 322 (33) | 321 (34) | 328 (27) | 328 (27) | 333 (22) | 332 (23) | 333 (22) | 333 (22) | 319 (36) | 333 (22) | 333 (22) | 333 (22) |
| **StrW** | 336 (19) | 336 (19) | 292 (63) | 291 (64) | 291 (64) | 322 (33) | 337 (18) | 337 (18) | 336 (19) | 331 (24) | 331 (24) | 337 (18) | 336 (19) | 337 (18) | 337 (18) | 323 (32) | 337 (18) | 336 (19) | 337 (18) |
| **StrC** | 336 (19) | 336 (19) | 292 (63) | 291 (64) | 291 (64) | 322 (33) | 337 (18) | 337 (18) | 336 (19) | 331 (24) | 331 (24) | 337 (18) | 336 (19) | 337 (18) | 337 (18) | 323 (32) | 337 (18) | 336 (19) | 337 (18) |
| **StrI** | 335 (20) | 335 (20) | 292 (63) | 291 (64) | 291 (64) | 321 (34) | 336 (19) | 336 (19) | 336 (19) | 330 (25) | 330 (25) | 336 (19) | 335 (20) | 336 (19) | 336 (19) | 323 (32) | 336 (19) | 335 (20) | 336 (19) |
| **LPS7** | 343 (12) | 343 (12) | 297 (58) | 296 (59) | 296 (59) | 328 (27) | 331 (24) | 331 (24) | 330 (25) | 344 (11) | 344 (11) | 343 (12) | 342 (13) | 343 (12) | 343 (12) | 329 (26) | 343 (12) | 342 (13) | 343 (12) |
| **LPS9** | 343 (12) | 343 (12) | 297 (58) | 296 (59) | 296 (59) | 328 (27) | 331 (24) | 331 (24) | 330 (25) | 344 (11) | 344 (11) | 343 (12) | 342 (13) | 343 (12) | 343 (12) | 329 (26) | 343 (12) | 342 (13) | 343 (12) |
| **SemWF** | 351 (4) | 351 (4) | 299 (56) | 298 (57) | 298 (57) | 333 (22) | 337 (18) | 337 (18) | 336 (19) | 343 (12) | 343 (12) | 354  (1) | 352 (3) | 354 (1) | 354 (1) | 338 (17) | 354 (1) | 353 (2) | 354 (1) |
| **PhoWF** | 350 (5) | 350 (5) | 298 (57) | 297 (58) | 297 (58) | 332 (23) | 336 (19) | 336 (19) | 335 (20) | 342 (13) | 342 (13) | 352  (3) | 352 (3) | 352 (3) | 352 (3) | 336 (19) | 352 (3) | 351 (4) | 352 (3) |
| **VbL** | 351 (4) | 351 (4) | 299 (56) | 298 (57) | 298 (57) | 333 (22) | 337 (18) | 337 (18) | 336 (19) | 343 (12) | 343 (12) | 354 (1) | 352 (3) | 354 (1) | 354 (1) | 338 (17) | 354 (1) | 353 (2) | 354 (1) |
| **VbR** | 351 (4) | 351 (4) | 299 (56) | 298 (57) | 298 (57) | 333 (22) | 337 (18) | 337 (18) | 336 (19) | 343 (12) | 343 (12) | 354  (1) | 352 (3) | 354 (1) | 354 (1) | 338 (17) | 354 (1) | 353 (2) | 354 (1) |
| **TB/A** | 336 (19) | 336 (19) | 293 (62) | 292 (63) | 292 (63) | 319 (36) | 323 (32) | 323 (32) | 323 (32) | 329 (26) | 329 (26) | 338 (17) | 336 (19) | 338 (17) | 338 (17) | 338 (17) | 338 (17) | 337 (18) | 338 (17) |
| **FigC** | 351 (4) | 351 (4) | 299 (56) | 298 (57) | 298 (57) | 333 (22) | 337 (18) | 337 (18) | 336 (19) | 343 (12) | 343 (12) | 354 (1) | 352  (3) | 354 (1) | 354 (1) | 338 (17) | 354 (1) | 353 (2) | 354 (1) |
| **FigR** | 350 (5) | 350 (5) | 298 (57) | 297 (58) | 297 (58) | 333 (22) | 336 (19) | 336 (19) | 335 (20) | 342 (13) | 342 (13) | 353  (2) | 351  (4) | 353 (2) | 353 (2) | 337 (18) | 353 (2) | 353 (2) | 353 (2) |
| **BNT** | 351 (4) | 351 (4) | 299 (56) | 298 (57) | 298 (57) | 333 (22) | 337 (18) | 337 (18) | 336 (19) | 343 (12) | 343 (12) | 354  (1) | 352  (3) | 354 (1) | 354 (1) | 338 (17) | 354 (1) | 353 (2) | 354 (1) |

*Note.* Values are valid (missing) pairwise-observations for each variable combination.
Abbreviations: BNT, Boston Naming Test; BTA, Brief Test of Attention; CScat, Modified Card Sorting Test categories; CSnpe, Modified Card Sorting Test non-perservative errors; CSpe, Modified Card Sorting Test perservative errors; DSbw, Digit Span backwards; DSfw, Digit Span forward; EGA, exploratory graph analysis; FigC, Figures Copy; FigR, Figures Recall; LPS7, Leistungsprüfsystem 7; LPS9, Leistungsprüfsystem 9; PhoWF, phonematic Word Fluency; SemWF, semantic Word Fluency; StrC, Stroop color naming; StrI, Stroop interference; StrW, Stroop word reading; TB/A, Trail Making Test B/A; VbL, Verbal Learning; VbR, Verbal Recall.

**Table S11***. Valid and missing pairwise-observations at assessment 12 months after baseline (t1).*

| **Variable** | **DSfw** | **DSbw** | **CScat** | **CSnpe** | **CSpe** | **BTA** | **StrW** | **StrC** | **StrI** | **LPS7** | **LPS9** | **SemWF** | **PhoWF** | **VbL** | **VbR** | **TB/A** | **FigC** | **FigR** | **BNT** |
| --- | --- | --- | --- | --- | --- | --- | --- | --- | --- | --- | --- | --- | --- | --- | --- | --- | --- | --- | --- |
| **DSfw** | 331 (24) | 331 (24) | 273 (82) | 273 (82) | 273 (82) | 315 (40) | 314 (41) | 314 (41) | 314 (41) | 322 (33) | 319 (36) | 330 (25) | 329 (26) | 330 (25) | 329 (26) | 306 (49) | 327 (28) | 327 (28) | 328 (27) |
| **DSbw** | 331 (24) | 331 (24) | 273 (82) | 273 (82) | 273 (82) | 315 (40) | 314 (41) | 314 (41) | 314 (41) | 322 (33) | 319 (36) | 330 (25) | 329 (26) | 330 (25) | 329 (26) | 306 (49) | 327 (28) | 327 (28) | 328 (27) |
| **CScat** | 273 (82) | 273 (82) | 273 (82) | 273 (82) | 273 (82) | 270 (85) | 267 (88) | 267 (88) | 267 (88) | 270 (85) | 268 (87) | 273 (82) | 272 (83) | 273 (82) | 272 (83) | 260 (95) | 273 (82) | 273 (82) | 272 (83) |
| **CSnpe** | 273 (82) | 273 (82) | 273 (82) | 273 (82) | 273 (82) | 270 (85) | 267 (88) | 267 (88) | 267 (88) | 270 (85) | 268 (87) | 273 (82) | 272 (83) | 273 (82) | 272 (83) | 260 (95) | 273 (82) | 273 (82) | 272 (83) |
| **CSpe** | 273 (82) | 273 (82) | 273 (82) | 273 (82) | 273 (82) | 270 (85) | 267 (88) | 267 (88) | 267 (88) | 270 (85) | 268 (87) | 273 (82) | 272 (83) | 273 (82) | 272 (83) | 260 (95) | 273 (82) | 273 (82) | 272 (83) |
| **BTA** | 315 (40) | 315 (40) | 270 (85) | 270 (85) | 270 (85) | 315 (40) | 304 (51) | 304 (51) | 304 (51) | 310 (45) | 308 (47) | 315 (40) | 314 (41) | 315 (40) | 314 (41) | 294 (61) | 312 (43) | 312 (43) | 314 (41) |
| **StrW** | 314 (41) | 314 (41) | 267 (88) | 267 (88) | 267 (88) | 304 (51) | 315 (40) | 315 (40) | 315 (40) | 309 (46) | 308 (47) | 314 (41) | 313 (42) | 314 (41) | 313 (42) | 297 (58) | 312 (43) | 312 (43) | 312 (43) |
| **StrC** | 314 (41) | 314 (41) | 267 (88) | 267 (88) | 267 (88) | 304 (51) | 315 (40) | 315 (40) | 315 (40) | 309 (46) | 308 (47) | 314 (41) | 313 (42) | 314 (41) | 313 (42) | 297 (58) | 312 (43) | 312 (43) | 312 (43) |
| **StrI** | 314 (41) | 314 (41) | 267 (88) | 267 (88) | 267 (88) | 304 (51) | 315 (40) | 315 (40) | 315 (40) | 309 (46) | 308 (47) | 314 (41) | 313 (42) | 314 (41) | 313 (42) | 297 (58) | 312 (43) | 312 (43) | 312 (43) |
| **LPS7** | 322 (33) | 322 (33) | 270 (85) | 270 (85) | 270 (85) | 310 (45) | 309 (46) | 309 (46) | 309 (46) | 322 (33) | 319 (36) | 321 (34) | 320 (35) | 321 (34) | 320 (35) | 300 (55) | 319 (36) | 319 (36) | 320 (35) |
| **LPS9** | 319 (36) | 319 (36) | 268 (87) | 268 (87) | 268 (87) | 308 (47) | 308 (47) | 308 (47) | 308 (47) | 319 (36) | 319 (36) | 318 (37) | 317 (38) | 318 (37) | 317 (38) | 298 (57) | 316 (39) | 316 (39) | 317 (38) |
| **SemWF** | 330 (25) | 330 (25) | 273 (82) | 273 (82) | 273 (82) | 315 (40) | 314 (41) | 314 (41) | 314 (41) | 321 (34) | 318 (37) | 338 (17) | 337 (18) | 338 (17) | 337 (18) | 313 (42) | 334 (21) | 333 (22) | 336 (19) |
| **PhoWF** | 329 (26) | 329 (26) | 272 (83) | 272 (83) | 272 (83) | 314 (41) | 313 (42) | 313 (42) | 313 (42) | 320 (35) | 317 (38) | 337 (18) | 337 (18) | 337 (18) | 336 (19) | 312 (43) | 333 (22) | 332 (23) | 335 (20) |
| **VbL** | 330 (25) | 330 (25) | 273 (82) | 273 (82) | 273 (82) | 315 (40) | 314 (41) | 314 (41) | 314 (41) | 321 (34) | 318 (37) | 338 (17) | 337 (18) | 338 (17) | 337 (18) | 313 (42) | 334 (21) | 333 (22) | 336 (19) |
| **VbR** | 329 (26) | 329 (26) | 272 (83) | 272 (83) | 272 (83) | 314 (41) | 313 (42) | 313 (42) | 313 (42) | 320 (35) | 317 (38) | 337 (18) | 336 (19) | 337 (18) | 337 (18) | 312 (43) | 333 (22) | 332 (23) | 335 (20) |
| **TB/A** | 306 (49) | 306 (49) | 260 (95) | 260 (95) | 260 (95) | 294 (61) | 297 (58) | 297 (58) | 297 (58) | 300 (55) | 298 (57) | 313 (42) | 312 (43) | 313 (42) | 312 (43) | 313 (42) | 311 (44) | 311 (44) | 311 (44) |
| **FigC** | 327 (28) | 327 (28) | 273 (82) | 273 (82) | 273 (82) | 312 (43) | 312 (43) | 312 (43) | 312 (43) | 319 (36) | 316 (39) | 334 (21) | 333 (22) | 334 (21) | 333 (22) | 311 (44) | 334 (21) | 332 (23) | 332 (23) |
| **FigR** | 327 (28) | 327 (28) | 273 (82) | 273 (82) | 273 (82) | 312 (43) | 312 (43) | 312 (43) | 312 (43) | 319 (36) | 316 (39) | 333 (22) | 332 (23) | 333 (22) | 332 (23) | 311 (44) | 332 (23) | 333 (22) | 331 (24) |
| **BNT** | 328 (27) | 328 (27) | 272 (83) | 272 (83) | 272 (83) | 314 (41) | 312 (43) | 312 (43) | 312 (43) | 320 (35) | 317 (38) | 336 (19) | 335 (20) | 336 (19) | 335 (20) | 311 (44) | 332 (23) | 331 (24) | 336 (19) |

*Note.* Values are valid (missing) pairwise-observations for each variable combination.
Abbreviations: BNT, Boston Naming Test; BTA, Brief Test of Attention; CScat, Modified Card Sorting Test categories; CSnpe, Modified Card Sorting Test non-perservative errors; CSpe, Modified Card Sorting Test perservative errors; DSbw, Digit Span backwards; DSfw, Digit Span forward; EGA, exploratory graph analysis; FigC, Figures Copy; FigR, Figures Recall; LPS7, Leistungsprüfsystem 7; LPS9, Leistungsprüfsystem 9; PhoWF, phonematic Word Fluency; SemWF, semantic Word Fluency; StrC, Stroop color naming; StrI, Stroop interference; StrW, Stroop word reading; TB/A, Trail Making Test B/A; VbL, Verbal Learning; VbR, Verbal Recall.

**Table S12***. Valid and missing pairwise-observations at assessment 24 months after baseline (t2).*

| **Variable** | **DSfw** | **DSbw** | **CScat** | **CSnpe** | **CSpe** | **BTA** | **StrW** | **StrC** | **StrI** | **LPS7** | **LPS9** | **SemWF** | **PhoWF** | **VbL** | **VbR** | **TB/A** | **FigC** | **FigR** | **BNT** |
| --- | --- | --- | --- | --- | --- | --- | --- | --- | --- | --- | --- | --- | --- | --- | --- | --- | --- | --- | --- |
| **DSfw** | 325 (30) | 325 (30) | 248 (107) | 248 (107) | 248 (107) | 310 (45) | 302 (53) | 302 (53) | 301 (54) | 307 (48) | 308 (47) | 325 (30) | 324 (31) | 325 (30) | 325 (30) | 296 (59) | 323 (32) | 323 (32) | 324 (31) |
| **DSbw** | 325 (30) | 326 (29) | 248 (107) | 248 (107) | 248 (107) | 310 (45) | 302 (53) | 302 (53) | 301 (54) | 307 (48) | 308 (47) | 326 (29) | 325 (30) | 326 (29) | 326 (29) | 297 (58) | 324 (31) | 324 (31) | 325 (30) |
| **CScat** | 248 (107) | 248 (107) | 249 (106) | 249 (106) | 249 (106) | 242 (113) | 242 (113) | 242 (113) | 242 (113) | 241 (114) | 242 (113) | 249 (106) | 248 (107) | 249 (106) | 249 (106) | 237 (118) | 249 (106) | 249 (106) | 249 (106) |
| **CSnpe** | 248 (107) | 248 (107) | 249 (106) | 249 (106) | 249 (106) | 242 (113) | 242 (113) | 242 (113) | 242 (113) | 241 (114) | 242 (113) | 249 (106) | 248 (107) | 249 (106) | 249 (106) | 237 (118) | 249 (106) | 249 (106) | 249 (106) |
| **CSpe** | 248 (107) | 248 (107) | 249 (106) | 249 (106) | 249 (106) | 242 (113) | 242 (113) | 242 (113) | 242 (113) | 241 (114) | 242 (113) | 249 (106) | 248 (107) | 249 (106) | 249 (106) | 237 (118) | 249 (106) | 249 (106) | 249 (106) |
| **BTA** | 310 (45) | 310 (45) | 242 (113) | 242 (113) | 242 (113) | 315 (40) | 298 (57) | 298 (57) | 297 (58) | 298 (57) | 299 (56) | 314 (41) | 313 (42) | 314 (41) | 314 (41) | 290 (65) | 312 (43) | 312 (43) | 313 (42) |
| **StrW** | 302 (53) | 302 (53) | 242 (113) | 242 (113) | 242 (113) | 298 (57) | 306 (49) | 306 (49) | 305 (50) | 294 (61) | 294 (61) | 305 (50) | 304 (51) | 305 (50) | 305 (50) | 287 (68) | 305 (50) | 305 (50) | 304 (51) |
| **StrC** | 302 (53) | 302 (53) | 242 (113) | 242 (113) | 242 (113) | 298 (57) | 306 (49) | 306 (49) | 305 (50) | 294 (61) | 294 (61) | 305 (50) | 304 (51) | 305 (50) | 305 (50) | 287 (68) | 305 (50) | 305 (50) | 304 (51) |
| **StrI** | 301 (54) | 301 (54) | 242 (113) | 242 (113) | 242 (113) | 297 (58) | 305 (50) | 305 (50) | 305 (50) | 293 (62) | 293 (62) | 304 (51) | 303 (52) | 304 (51) | 304 (51) | 287 (68) | 304 (51) | 304 (51) | 303 (52) |
| **LPS7** | 307 (48) | 307 (48) | 241 (114) | 241 (114) | 241 (114) | 298 (57) | 294 (61) | 294 (61) | 293 (62) | 309 (46) | 308 (47) | 309 (46) | 308 (47) | 309 (46) | 309 (46) | 290 (65) | 309 (46) | 309 (46) | 308 (47) |
| **LPS9** | 308 (47) | 308 (47) | 242 (113) | 242 (113) | 242 (113) | 299 (56) | 294 (61) | 294 (61) | 293 (62) | 308 (47) | 310 (45) | 310 (45) | 309 (46) | 310 (45) | 310 (45) | 289 (66) | 310 (45) | 310 (45) | 309 (46) |
| **SemWF** | 325 (30) | 326 (29) | 249 (106) | 249 (106) | 249 (106) | 314 (41) | 305 (50) | 305 (50) | 304 (51) | 309 (46) | 310 (45) | 335 (20) | 334 (21) | 334 (21) | 334 (21) | 303 (52) | 332 (23) | 332 (23) | 333 (22) |
| **PhoWF** | 324 (31) | 325 (30) | 248 (107) | 248 (107) | 248 (107) | 313 (42) | 304 (51) | 304 (51) | 303 (52) | 308 (47) | 309 (46) | 334 (21) | 334 (21) | 333 (22) | 333 (22) | 302 (53) | 331 (24) | 331 (24) | 332 (23) |
| **VbL** | 325 (30) | 326 (29) | 249 (106) | 249 (106) | 249 (106) | 314 (41) | 305 (50) | 305 (50) | 304 (51) | 309 (46) | 310 (45) | 334 (21) | 333 (22) | 334 (21) | 334 (21) | 302 (53) | 332 (23) | 332 (23) | 332 (23) |
| **VbR** | 325 (30) | 326 (29) | 249 (106) | 249 (106) | 249 (106) | 314 (41) | 305 (50) | 305 (50) | 304 (51) | 309 (46) | 310 (45) | 334 (21) | 333 (22) | 334 (21) | 334 (21) | 302 (53) | 332 (23) | 332 (23) | 332 (23) |
| **TB/A** | 296 (59) | 297 (58) | 237 (118) | 237 (118) | 237 (118) | 290 (65) | 287 (68) | 287 (68) | 287 (68) | 290 (65) | 289 (66) | 303 (52) | 302 (53) | 302 (53) | 302 (53) | 304 (51) | 302 (53) | 302 (53) | 301 (54) |
| **FigC** | 323 (32) | 324 (31) | 249 (106) | 249 (106) | 249 (106) | 312 (43) | 305 (50) | 305 (50) | 304 (51) | 309 (46) | 310 (45) | 332 (23) | 331 (24) | 332 (23) | 332 (23) | 302 (53) | 332 (23) | 332 (23) | 330 (25) |
| **FigR** | 323 (32) | 324 (31) | 249 (106) | 249 (106) | 249 (106) | 312 (43) | 305 (50) | 305 (50) | 304 (51) | 309 (46) | 310 (45) | 332 (23) | 331 (24) | 332 (23) | 332 (23) | 302 (53) | 332 (23) | 332 (23) | 330 (25) |
| **BNT** | 324 (31) | 325 (30) | 249 (106) | 249 (106) | 249 (106) | 313 (42) | 304 (51) | 304 (51) | 303 (52) | 308 (47) | 309 (46) | 333 (22) | 332 (23) | 332 (23) | 332 (23) | 301 (54) | 330 (25) | 330 (25) | 333 (22) |

*Note.* Values are valid (missing) pairwise-observations for each variable combination.
Abbreviations: BNT, Boston Naming Test; BTA, Brief Test of Attention; CScat, Modified Card Sorting Test categories; CSnpe, Modified Card Sorting Test non-perservative errors; CSpe, Modified Card Sorting Test perservative errors; DSbw, Digit Span backwards; DSfw, Digit Span forward; EGA, exploratory graph analysis; FigC, Figures Copy; FigR, Figures Recall; LPS7, Leistungsprüfsystem 7; LPS9, Leistungsprüfsystem 9; PhoWF, phonematic Word Fluency; SemWF, semantic Word Fluency; StrC, Stroop color naming; StrI, Stroop interference; StrW, Stroop word reading; TB/A, Trail Making Test B/A; VbL, Verbal Learning; VbR, Verbal Recall.

**Table S13***. Valid and missing pairwise-observations at assessment 36 months after baseline (t3).*

| **Variable** | **DSfw** | **DSbw** | **CScat** | **CSnpe** | **CSpe** | **BTA** | **StrW** | **StrC** | **StrI** | **LPS7** | **LPS9** | **SemWF** | **PhoWF** | **VbL** | **VbR** | **TB/A** | **FigC** | **FigR** | **BNT** |
| --- | --- | --- | --- | --- | --- | --- | --- | --- | --- | --- | --- | --- | --- | --- | --- | --- | --- | --- | --- |
| **DSfw** | 338 (17) | 336 (19) | 226 (129) | 226 (129) | 226 (129) | 303 (52) | 307 (48) | 306 (49) | 305 (50) | 314 (41) | 321 (34) | 338 (17) | 338 (17) | 338 (17) | 338 (17) | 308 (47) | 336 (19) | 335 (20) | 338 (17) |
| **DSbw** | 336 (19) | 336 (19) | 226 (129) | 226 (129) | 226 (129) | 303 (52) | 306 (49) | 305 (50) | 304 (51) | 314 (41) | 320 (35) | 336 (19) | 336 (19) | 336 (19) | 336 (19) | 308 (47) | 334 (21) | 333 (22) | 336 (19) |
| **CScat** | 226 (129) | 226 (129) | 230 (125) | 230 (125) | 230 (125) | 216 (139) | 219 (136) | 218 (137) | 218 (137) | 222 (133) | 226 (129) | 230 (125) | 230 (125) | 230 (125) | 230 (125) | 216 (139) | 229 (126) | 229 (126) | 230 (125) |
| **CSnpe** | 226 (129) | 226 (129) | 230 (125) | 230 (125) | 230 (125) | 216 (139) | 219 (136) | 218 (137) | 218 (137) | 222 (133) | 226 (129) | 230 (125) | 230 (125) | 230 (125) | 230 (125) | 216 (139) | 229 (126) | 229 (126) | 230 (125) |
| **CSpe** | 226 (129) | 226 (129) | 230 (125) | 230 (125) | 230 (125) | 216 (139) | 219 (136) | 218 (137) | 218 (137) | 222 (133) | 226 (129) | 230 (125) | 230 (125) | 230 (125) | 230 (125) | 216 (139) | 229 (126) | 229 (126) | 230 (125) |
| **BTA** | 303 (52) | 303 (52) | 216 (139) | 216 (139) | 216 (139) | 306 (49) | 294 (61) | 293 (62) | 292 (63) | 295 (60) | 301 (54) | 306 (49) | 306 (49) | 306 (49) | 306 (49) | 285 (70) | 304 (51) | 303 (52) | 306 (49) |
| **StrW** | 307 (48) | 306 (49) | 219 (136) | 219 (136) | 219 (136) | 294 (61) | 311 (44) | 310 (45) | 309 (46) | 300 (55) | 306 (49) | 311 (44) | 311 (44) | 311 (44) | 311 (44) | 292 (63) | 309 (46) | 308 (47) | 311 (44) |
| **StrC** | 306 (49) | 305 (50) | 218 (137) | 218 (137) | 218 (137) | 293 (62) | 310 (45) | 310 (45) | 309 (46) | 299 (56) | 305 (50) | 310 (45) | 310 (45) | 310 (45) | 310 (45) | 291 (64) | 308 (47) | 307 (48) | 310 (45) |
| **StrI** | 305 (50) | 304 (51) | 218 (137) | 218 (137) | 218 (137) | 292 (63) | 309 (46) | 309 (46) | 309 (46) | 298 (57) | 304 (51) | 309 (46) | 309 (46) | 309 (46) | 309 (46) | 291 (64) | 307 (48) | 306 (49) | 309 (46) |
| **LPS7** | 314 (41) | 314 (41) | 222 (133) | 222 (133) | 222 (133) | 295 (60) | 300 (55) | 299 (56) | 298 (57) | 318 (37) | 318 (37) | 318 (37) | 318 (37) | 318 (37) | 318 (37) | 296 (59) | 317 (38) | 316 (39) | 318 (37) |
| **LPS9** | 321 (34) | 320 (35) | 226 (129) | 226 (129) | 226 (129) | 301 (54) | 306 (49) | 305 (50) | 304 (51) | 318 (37) | 325 (30) | 325 (30) | 325 (30) | 325 (30) | 325 (30) | 299 (56) | 324 (31) | 323 (32) | 325 (30) |
| **SemWF** | 338 (17) | 336 (19) | 230 (125) | 230 (125) | 230 (125) | 306 (49) | 311 (44) | 310 (45) | 309 (46) | 318 (37) | 325 (30) | 349  (6) | 349 (6) | 349 (6) | 348 (7) | 316 (39) | 346 (9) | 345 (10) | 349 (6) |
| **PhoWF** | 338 (17) | 336 (19) | 230 (125) | 230 (125) | 230 (125) | 306 (49) | 311 (44) | 310 (45) | 309 (46) | 318 (37) | 325 (30) | 349 (6) | 349 (6) | 349 (6) | 348 (7) | 316 (39) | 346 (9) | 345 (10) | 349 (6) |
| **VbL** | 338 (17) | 336 (19) | 230 (125) | 230 (125) | 230 (125) | 306 (49) | 311 (44) | 310 (45) | 309 (46) | 318 (37) | 325 (30) | 349  (6) | 349 (6) | 349 (6) | 348 (7) | 316 (39) | 346 (9) | 345 (10) | 349 (6) |
| **VbR** | 338 (17) | 336 (19) | 230 (125) | 230 (125) | 230 (125) | 306 (49) | 311 (44) | 310 (45) | 309 (46) | 318 (37) | 325 (30) | 348  (7) | 348 (7) | 348 (7) | 348 (7) | 316 (39) | 346 (9) | 345 (10) | 348 (7) |
| **TB/A** | 308 (47) | 308 (47) | 216 (139) | 216 (139) | 216 (139) | 285 (70) | 292 (63) | 291 (64) | 291 (64) | 296 (59) | 299 (56) | 316 (39) | 316 (39) | 316 (39) | 316 (39) | 316 (39) | 315 (40) | 314 (41) | 316 (39) |
| **FigC** | 336 (19) | 334 (21) | 229 (126) | 229 (126) | 229 (126) | 304 (51) | 309 (46) | 308 (47) | 307 (48) | 317 (38) | 324 (31) | 346 (9) | 346 (9) | 346 (9) | 346 (9) | 315 (40) | 346 (9) | 345 (10) | 346 (9) |
| **FigR** | 335 (20) | 333 (22) | 229 (126) | 229 (126) | 229 (126) | 303 (52) | 308 (47) | 307 (48) | 306 (49) | 316 (39) | 323 (32) | 345 (10) | 345 (10) | 345 (10) | 345 (10) | 314 (41) | 345 (10) | 345 (10) | 345 (10) |
| **BNT** | 338 (17) | 336 (19) | 230 (125) | 230 (125) | 230 (125) | 306 (49) | 311 (44) | 310 (45) | 309 (46) | 318 (37) | 325 (30) | 349  (6) | 349 (6) | 349 (6) | 348 (7) | 316 (39) | 346 (9) | 345 (10) | 349 (6) |

*Note.* Values are valid (missing) pairwise-observations for each variable combination.
Abbreviations: BNT, Boston Naming Test; BTA, Brief Test of Attention; CScat, Modified Card Sorting Test categories; CSnpe, Modified Card Sorting Test non-perservative errors; CSpe, Modified Card Sorting Test perservative errors; DSbw, Digit Span backwards; DSfw, Digit Span forward; EGA, exploratory graph analysis; FigC, Figures Copy; FigR, Figures Recall; LPS7, Leistungsprüfsystem 7; LPS9, Leistungsprüfsystem 9; PhoWF, phonematic Word Fluency; SemWF, semantic Word Fluency; StrC, Stroop color naming; StrI, Stroop interference; StrW, Stroop word reading; TB/A, Trail Making Test B/A; VbL, Verbal Learning; VbR, Verbal Recall.

**Table S14***. Descriptive cognitive test scores of the total sample (N = 355) for each time point of assessment.*

| **Test score** | **t0** | **t1** | **t2** | **t3** |
| --- | --- | --- | --- | --- |
| Digit Span forward | 0.18 (0.97) | 0.16 (0.97) | 0.12 (1.02) | 0.02 (0.98) |
| Brief Test of Attention | 0.00 (1.16) | -0.03 (1.23) | -0.09 (1.30) | -0.16 (1.27) |
| Stroop words reading | 0.37 (0.81) | 0.38 (0.80) | 0.32 (0.79) | 0.32 (0.83) |
| Stroop color naming | 0.33 (0.94) | 0.34 (0.91) | 0.29 (0.93) | 0.26 (1.00) |
| Stroop interference | 0.56 (0.84) | 0.58 (0.85) | 0.61 (0.80) | 0.62 (0.90) |
| CERAD+ Semantic Word Fluency | -0.11 (1.18) | -0.20 (1.18) | -0.22 (1.24) | -0.31 (1.26) |
| CERAD+ Phonematic Word Fluency | 0.18 (1.17) | 0.18 (1.15) | 0.17 (1.23) | 0.09 (1.29) |
| CERAD+ Trail Making Test B/A | -0.18 (1.05) | -0.16 (1.05) | -0.17 (1.11) | -0.29 (1.14) |
| Digit Span backwards | -0.18 (0.99) | -0.22 (0.94) | -0.32 (0.97) | -0.38 (0.94) |
| Modified Card Sorting Test categories | -0.68 (1.22) | -0.72 (1.27) | -0.67 (1.16) | -0.87 (1.34) |
| Modified Card Sorting Test non-perservative errors | -0.43 (0.95) | -0.39 (0.93) | -0.40 (0.89) | -0.42 (0.87) |
| Modified Card Sorting Test perservative errors | -0.26 (0.77) | -0.23 (0.85) | -0.26 (0.77) | -0.38 (0.86) |
| CERAD+ Figures Recall | -0.24 (1.40) | -0.26 (1.34) | -0.11 (1.35) | -0.29 (1.42) |
| CERAD+ Verbal Learning | -0.41 (1.33) | -0.19 (1.32) | -0.22 (1.45) | -0.29 (1.55) |
| CERAD+ Verbal Recall | -0.19 (1.14) | -0.10 (1.17) | -0.14 (1.18) | -0.14 (1.21) |
| CERAD+ Boston Naming Test | 0.34 (0.92) | 0.30 (0.92) | 0.25 (0.95) | 0.24 (0.98) |
| CERAD+ Figures Copy | -0.20 (1.35) | -0.17 (1.27) | -0.03 (1.21) | -0.21 (1.29) |
| Leistungsprüfsystem 7 | 0.03 (1.12) | 0.07 (1.14) | 0.08 (1.15) | -0.01 (1.15) |
| Leistungsprüfsystem 9 | 0.42 (0.95) | 0.42 (1.02) | 0.46 (0.99) | 0.47 (0.99) |

*Note*. Descriptive cognitive test scores for each time point of assessment of the total sample included in analyses (N = 355). Data are mean (standard deviation).

**Table S15***. Descriptive cognitive test scores of the subgroup of people with Parkinson’s disease and normal cognition (n = 185) for each time point of assessment.*

| **Test score** | **t0** | **t1** | **t2** | **t3** |
| --- | --- | --- | --- | --- |
| Digit Span forward | 0.34 (0.98) | 0.34 (0.97) | 0.35 (0.97) | 0.25 (0.94) |
| Brief Test of Attention | 0.38 (0.82) | 0.35 (0.87) | 0.40 (0.86) | 0.22 (1.05) |
| Stroop words reading | 0.59 (0.73) | 0.53 (0.75) | 0.52 (0.71) | 0.55 (0.74) |
| Stroop color naming | 0.59 (0.88) | 0.60 (0.85) | 0.57 (0.84) | 0.58 (0.92) |
| Stroop interference | 0.75 (0.81) | 0.82 (0.69) | 0.84 (0.70) | 0.86 (0.74) |
| CERAD+ Semantic Word Fluency | 0.21 (1.09) | 0.05 (1.19) | 0.15 (1.13) | 0.08 (1.20) |
| CERAD+ Phonematic Word Fluency | 0.54 (1.02) | 0.50 (1.05) | 0.60 (1.08) | 0.50 (1.13) |
| CERAD+ Trail Making Test B/A | -0.04 (0.98) | -0.12 (1.01) | -0.01 (1.01) | -0.11 (1.11) |
| Digit Span backwards | 0.11 (0.97) | 0.01 (0.91) | 0.02 (0.87) | -0.06 (0.86) |
| Modified Card Sorting Test categories | -0.18 (0.81) | -0.40 (1.10) | -0.33 (0.99) | -0.49 (1.19) |
| Modified Card Sorting Test non-perservative errors | -0.15 (0.69) | -0.18 (0.83) | -0.19 (0.85) | -0.17 (0.76) |
| Modified Card Sorting Test perservative errors | -0.07 (0.49) | -0.05 (0.66) | -0.07 (0.58) | -0.20 (0.67) |
| CERAD+ Figures Recall | 0.21 (1.17) | 0.17 (1.15) | 0.24 (1.19) | 0.21 (1.23) |
| CERAD+ Verbal Learning | 0.11 (0.94) | 0.41 (1.07) | 0.32 (1.20) | 0.39 (1.12) |
| CERAD+ Verbal Recall | 0.16 (0.98) | 0.34 (0.97) | 0.30 (1.04) | 0.35 (1.02) |
| CERAD+ Boston Naming Test | 0.52 (0.71) | 0.55 (0.68) | 0.47 (0.74) | 0.53 (0.72) |
| CERAD+ Figures Copy | 0.18 (1.04) | 0.23 (0.94) | 0.30 (0.90) | 0.25 (0.92) |
| Leistungsprüfsystem 7 | 0.47 (0.92) | 0.43 (1.07) | 0.43 (1.08) | 0.39 (0.98) |
| Leistungsprüfsystem 9 | 0.74 (0.86) | 0.76 (0.96) | 0.83 (0.94) | 0.82 (0.89) |

*Note*. Descriptive cognitive test scores for each time point of assessment of the subgroup of people with Parkinson’s disease and normal cognition (N = 185). Data are mean (standard deviation).

**Table S16***. Descriptive cognitive test scores of the subgroup of people with Parkinson’s disease and mild cognitive impairment (n = 145) for each time point of assessment.*

| **Test score** | **t0** | **t1** | **t2** | **t3** |
| --- | --- | --- | --- | --- |
| Digit Span forward | 0.01 (0.94) | -0.02 (0.99) | -0.10 (1.01) | -0.17 (0.96) |
| Brief Test of Attention | -0.24 (1.21) | -0.27 (1.29) | -0.38 (1.30) | -0.52 (1.24) |
| Stroop words reading | 0.22 (0.75) | 0.27 (0.81) | 0.16 (0.77) | 0.10 (0.83) |
| Stroop color naming | 0.14 (0.87) | 0.14 (0.85) | 0.07 (0.85) | -0.07 (0.92) |
| Stroop interference | 0.47 (0.76) | 0.45 (0.78) | 0.45 (0.72) | 0.38 (0.94) |
| CERAD+ Semantic Word Fluency | -0.36 (1.12) | -0.36 (1.07) | -0.46 (1.21) | -0.62 (1.19) |
| CERAD+ Phonematic Word Fluency | -0.08 (1.14) | -0.04 (1.10) | -0.12 (1.15) | -0.24 (1.26) |
| CERAD+ Trail Making Test B/A | -0.36 (1.08) | -0.23 (1.08) | -0.39 (1.18) | -0.60 (1.09) |
| Digit Span backwards | -0.44 (0.91) | -0.42 (0.91) | -0.58 (0.92) | -0.66 (0.87) |
| Modified Card Sorting Test categories | -1.23 (1.40) | -1.00 (1.34) | -0.97 (1.23) | -1.23 (1.39) |
| Modified Card Sorting Test non-perservative errors | -0.79 (1.11) | -0.59 (1.00) | -0.61 (0.88) | -0.69 (0.90) |
| Modified Card Sorting Test perservative errors | -0.43 (0.90) | -0.38 (0.98) | -0.39 (0.84) | -0.51 (0.97) |
| CERAD+ Figures Recall | -0.54 (1.42) | -0.66 (1.41) | -0.33 (1.40) | -0.64 (1.38) |
| CERAD+ Verbal Learning | -0.79 (1.29) | -0.74 (1.25) | -0.60 (1.37) | -0.85 (1.50) |
| CERAD+ Verbal Recall | -0.44 (1.09) | -0.47 (1.14) | -0.44 (1.04) | -0.60 (1.17) |
| CERAD+ Boston Naming Test | 0.31 (0.90) | 0.14 (0.95) | 0.09 (1.03) | 0.09 (1.02) |
| CERAD+ Figures Copy | -0.42 (1.38) | -0.46 (1.36) | -0.27 (1.36) | -0.49 (1.29) |
| Leistungsprüfsystem 7 | -0.31 (1.11) | -0.26 (1.04) | -0.20 (1.09) | -0.42 (1.18) |
| Leistungsprüfsystem 9 | 0.20 (0.85) | 0.08 (0.91) | 0.12 (0.86) | 0.14 (0.95) |

*Note*. Descriptive cognitive test scores for each time point of assessment of the subgroup of people with Parkinson’s disease with mild cognitive impairment (N = 145). Data are mean (standard deviation).

**Table S17***. Descriptive cognitive test scores of the subgroup of people with Parkinson’s disease and dementia (n = 25) for each time point of assessment.*

| **Test score** | **t0** | **t1** | **t2** | **t3** |
| --- | --- | --- | --- | --- |
| Digit Span forward | -0.01 (0.85) | -0.21 (0.93) | -0.30 (1.00) | -0.59 (0.97) |
| Brief Test of Attention | -1.57 (1.46) | -1.66 (1.68) | -2.21 (1.60) | -1.66 (1.95) |
| Stroop words reading | -0.58 (0.93) | -0.34 (0.78) | -0.67 (1.04) | -0.67 (0.85) |
| Stroop color naming | -0.65 (0.96) | -0.66 (0.89) | -1.08 (0.91) | -0.85 (1.27) |
| Stroop interference | -0.40 (0.98) | -0.74 (1.29) | -0.89 (0.86) | -0.50 (1.25) |
| CERAD+ Semantic Word Fluency | -1.02 (1.33) | -1.22 (0.99) | -1.56 (0.91) | -1.53 (0.83) |
| CERAD+ Phonematic Word Fluency | -0.95 (1.23) | -0.89 (1.23) | -1.33 (1.18) | -1.13 (1.35) |
| CERAD+ Trail Making Test B/A | -0.26 (1.31) | 0.03 (1.39) | -0.19 (1.42) | 0.38 (1.56) |
| Digit Span backwards | -0.86 (0.93) | -0.87 (0.84) | -1.34 (0.73) | -1.21 (0.99) |
| Modified Card Sorting Test categories | -1.56 (0.86) | -1.73 (1.24) | -1.91 (0.69) | 2.30 (0.53) |
| Modified Card Sorting Test non-perservative errors | -0.47 (1.12) | -0.85 (0.79) | -0.79 (0.94) | -0.95 (0.82) |
| Modified Card Sorting Test perservative errors | -1.20 (1.25) | -0.79 (0.98) | -1.22 (1.07) | -1.64 (0.63) |
| CERAD+ Figures Recall | -1.86 (1.22) | -1.32 (0.81) | -1.45 (1.02) | -1.98 (1.08) |
| CERAD+ Verbal Learning | -2.07 (1.88) | -1.52 (1.03) | -2.00 (1.49) | -2.16 (1.81) |
| CERAD+ Verbal Recall | -1.25 (1.48) | -1.30 (1.09) | -1.56 (1.27) | -1.22 (1.14) |
| CERAD+ Boston Naming Test | -0.91 (1.39) | -0.70 (1.29) | -0.44 (1.25) | -1.01 (1.33) |
| CERAD+ Figures Copy | -1.75 (1.79) | -1.56 (1.57) | -1.06 (1.44) | -2.19 (1.37) |
| Leistungsprüfsystem 7 | -1.28 (0.76) | -0.88 (1.06) | -1.13 (0.89) | -0.95 (0.91) |
| Leistungsprüfsystem 9 | -0.74 (0.93) | -0.53 (0.90) | -0.50 (0.82) | -0.53 (0.58) |

*Note*. Descriptive cognitive test scores for each time point of assessment of the subgroup of people with Parkinson’s disease with dementia (N = 25). Data are mean (standard deviation).
